# Lecithin:cholesterol acyltransferase binds a discontinuous binding site on adjacent apolipoprotein A-I belts in HDL

**DOI:** 10.1016/j.jlr.2025.100786

**Published:** 2025-03-25

**Authors:** Bethany Coleman, Shimpi Bedi, John H. Hill, Jamie Morris, Kelly A. Manthei, Rachel C. Hart, Yi He, Amy S. Shah, W. Gray Jerome, Tomas Vaisar, Karin E. Bornfeldt, Hyun Song, Jere P. Segrest, Jay W. Heinecke, Stephen G. Aller, John J.G. Tesmer, W. Sean Davidson

**Affiliations:** 1 Department of Molecular and Cellular Biosciences, University of Cincinnati, Cincinnati, OH, USA; 2 Department of Pathology and Laboratory Medicine, University of Cincinnati, Cincinnati, OH, USA; 3 Department of Pharmacology and Toxicology, University of Alabama at Birmingham, Birmingham, AL, USA; 4 Life Sciences Institute, University of Michigan, Ann Arbor, MI, USA; 5 Department of Pathology, Microbiology and Immunology, Vanderbilt University School of Medicine, Nashville, TN, USA; 6 Department of Medicine, University of Washington School of Medicine, Seattle, WA, USA; 7 Department of Pediatrics, Cincinnati Children’s Hospital Medical Center and the University of Cincinnati, Cincinnati, OH, USA; 8 Department of Medicine, Vanderbilt University Medical Center, Nashville, TN, USA; 9 Departments of Biological Sciences and Medicinal Chemistry and Molecular Pharmacology, Purdue University, West Lafayette, IN, USA

**Keywords:** apolipoprotein, apolipoprotein A-I, cholesterol esterification, cross-linking, cryo-electron microscopy, docking, HDL, LCAT, lecithin:cholesterol acyltransferase, mass spectrometry, mutagenesis, structure

## Abstract

Lecithin:cholesterol acyltransferase (LCAT) is a high-density lipoprotein (HDL) modifying protein that profoundly affects the composition and function of HDL subspecies. The cholesterol esterification activity of LCAT is dramatically increased by apolipoprotein A-I (APOA1) on HDL, but the mechanism remains unclear. Using site-directed mutagenesis, cross-linking, mass spectrometry, electron microscopy, protein engineering, and molecular docking, we identified two LCAT binding sites formed by helices 4 and 6 from two antiparallel APOA1 molecules in HDL. Although the reciprocating APOA1 “belts” form two ostensibly symmetrical binding locations, LCAT can adopt distinct orientations at each site, as shown by our 9.8 Å cryoEM envelope. In one case, LCAT membrane binding domains align with the APOA1 belts and, in the other, the HDL phospholipids. By introducing disulfide bonds between the APOA1 helical domains, we demonstrated that LCAT does not require helical separation during its reaction cycle. This indicates that LCAT, anchored to APOA1 belts, accesses substrates and deposits products through interactions with the planar lipid surface. This model of the LCAT/APOA1 interaction provides insights into how LCAT and possibly other HDL-modifying factors engage the APOA1 scaffold, offering potential strategies to enhance LCAT activity in individuals with genetic defects.

A strong case could be made for lecithin:cholesterol acyltransferase (LCAT) as the plasma factor that most profoundly affects lipoprotein metabolism. By virtue of its hydrolysis of phospholipid fatty acids and subsequent esterification to cholesterol, the enzyme is responsible for most of the cholesteryl ester (CE) generated in the plasma compartment (1). LCAT activity provides a concentration gradient for the movement of unesterified (free) cholesterol (FC) from peripheral cells into plasma and thus is an important component of reverse cholesterol transport (2, 3). Because CE comprises the majority of the neutral lipid core of high-density lipoproteins (HDL), LCAT plays a fundamental role in dictating HDL particle shape and size distribution (4). Given growing evidence that HDL is a family of diversely composed particles that play roles in a host of physiological functions, LCAT likely affects processes ranging from lipid transport to inflammation to immune responses (5, 6). Individuals with familial LCAT deficiency (FLD) lack plasma cholesterol esterification activity, exhibit low HDL cholesterol levels, and have elevated plasma FC and triglycerides (TG). The HDL particles they do possess tend to be small and discoidal (7, 8). Additionally, low density lipoprotein (LDL) cholesterol levels tend to be decreased in LCAT deficiency, skewing toward smaller TG-enriched particles, which are associated with atherosclerosis (9, 10). Finally, many FLD afflicted individuals exhibit large, abnormal vesicular structures of phospholipid and FC, called lipoprotein-X, that are likely responsible for accelerated renal disease seen in FLD (11, 12). Given its major role in both LDL and HDL metabolism, LCAT modulation therapies have been the subject of active investigation for preventing cardiovascular disease (13, 14) as well as potentially treating lipoprotein-X-induced kidney problems in LCAT deficient subjects (15, 16).

Due to its impact on lipoprotein metabolism and its therapeutic potential, it is important to understand the mechanistic basis of LCAT activation. Several HDL apolipoproteins can activate LCAT but apolipoprotein A-I (APOA1) is, by far, the most abundant and potent cofactor (17). A common theme of LCAT activators is the presence of amphipathic α-helices. For example, APOA1 is comprised of a string of 10 connected amphipathic α-helices that form anti-parallel belts that encircle the HDL particle (18). Even short amphipathic helical peptides can solubilize lipids and activate LCAT to various extents (19). Deletion and point mutagenesis experiments have shown that LCAT specifically interacts with APOA1 helices between amino acids 143 and 187 (extensively reviewed in (20)). Furthermore, we have shown that APOA1 may activate LCAT through a discontinuous binding site assembled across two opposing APOA1 helical belts composed of helix 4 of one APOA1 molecule and helix 6 of the other (21). Despite these advances, the molecular details underlying the binding and activation of LCAT remain unclear, largely from a lack of structural detail of the complex formed between LCAT and APOA1 on HDL. Previous work from our group used negative stain electron microscopy, chemical cross-linking, and hydrogen deuterium exchange to propose a model of how LCAT interacts with HDL (22). Here, we extend the resolution of the model by employing cryo-EM, alternative cross-linking strategies, and in silico docking analyses to propose the most detailed model for the LCAT/APOA1 interaction yet.

## Materials and methods

### Reagents used

Formaldehyde solution (37% formaldehyde) was purchased from Sigma-Aldrich. This was used at a final concentration of 2% formaldehyde in PBS (pH 7.3). Bis(sulfocuccinimidyl)suberate (BS^3^) and 1-ethyl-3-(3-dimethylaminopropyl)carbodiimide (EDC) were purchased from Thermo Scientific. EDC was solubilized in PBS (pH 6.5) to 0.1 M. BS^3^ was solubilized in PBS (pH 7.3) to 10 mg/ml. Recombinant LCAT used for all non-cryoEM experiments was gifted by Alan Remaley and was expressed in CHO cells. LCAT used in the cryoEM experiments was expressed in HEK293F cells as described below.

### Mutagenesis and preparation of APOA1 containing rHDL

Mutants of APOA1 were generated by site-directed mutagenesis as described (21, 23). Each mutant was expressed in *E. coli* and purified with an N-terminal 6x His-tag via nickel affinity chromatography as described previously (21). Reconstituted HDL particles were generated with 1-palmitoyl-2-oleoyl-sn-glycero-3-phosphocholine (POPC) and FC by cholate dialysis at a molar ratio of 85:5:1 (POPC:FC:APOA1) (21).

### Cross-linking rHDL-LCAT

For MS analysis, the rHDL:LCAT complex was prepared by mixing LCAT protein and APOA1-containing rHDL at a 3:1 ratio (LCAT:APOA1) in PBS. The sample was incubated at 37°C for 30 min. Formaldehyde cross-linking was initiated by mixing final concentrations of 2% formaldehyde and 20 mM protein in PBS (pH 7.3) then incubating the sample at room temperature (25°C) for 20 min (24). Reactions were quenched with 3 M ammonium bicarbonate (AB) to a final concentration of 1.5 M AB for 15 min at room temperature, fractionated by size exclusion chromatography, and processed as described below. BS^3^ cross-linking was initiated by adding 50:1 M ratio of BS^3^ to APOA1 in PBS (pH 7.3) then incubated for 2 h at 4°C. BS^3^ was quenched by 1M glycine to 200 mM final concentration glycine. EDC cross-linking was initiated by adding 100:1 M ratio of EDC to APOA1 in PBS (pH 6.5) then incubated for 2 h at 4°C. EDC was quenched by adding 1 M Tris HCl (pH 7.5) to a 200 mM final concentration. All crosslinking reactions were fractionated by size exclusion chromatography (triple Superdex 200 Increase (Cytiva)) and processed as described below.

### Mass spectrometry of cross-linked rHDL-LCAT

Purified cross-linked complexes were prepared by S-TRAP columns (Protifi, Fairport NY) according to the manufacturer’s protocol. The digestion was done overnight at 37°C with (1:10) mass ratio with trypsin. Formaldehyde cross-linked samples were prepared by a modified S-trap method including a room temperature reduction step and 2 h trypsin digestion. Protein digests were evaporated to dryness using a speed vac. In each experiment, 50–60 ng of digested cross-linked protein was analyzed by data-dependent acquisition LC-MS/MS. After desalting on a C18 trapping column (Reprosil-Pur 120 C18-AQ, 5 μm, 0.1 × 40 mm, Dr Maisch HPLC GmbH) (flow rate 4 μl/min), the digested peptides were separated on an analytical column (Reprosil-Pur 120 C18-AQ, 5 μm, 250 × 0.075 mm, Dr Maisch HPLC GmbH). The following multi-step linear gradient was used: 1–5%B in 2 min, 5%–25% in 50 min, 25%–35% in 10 min. At the end of the gradient column was washed with a ramp to 80%B and re-equilibrated (A - 0.1% formic acid in water, B - acetonitrile, 0.1% formic acid, flow rate of 0.4 μl/min). An LC-MSMS consisting of an easyLC 1,200 (Thermo Fisher), and a Thermo Orbitrap Exploris480 (Thermo Fisher) mass spectrometer with electrospray ionization were used for the analysis. Data dependent acquisition parameters were as follows: a full MS scan (m/z 300–1800) at 120,000 resolution and data-dependent HCD MS2 scans at resolution 30,000 on charge states 2–7 with high charge state priority excluding undetermined charge state precursors, dynamic exclusion of 30 s after acquisition of the MS2 scan, maximum ion time 54 ms, normalized AGC 400% and HCD normalized CE 28%.

### Identification of cross-linked peptides

pLink was used to identify putative cross-linked peptides using the following search parameters: 1% FDR and modifications were set to carbamidomethyl-Cys (mass shift 57.02146 Da) and oxidation of methionine (mass shift 15.9949 Da). For formaldehyde, the mass shift for cross-linked products was set to 24.000 Da, and modified residues were set to Lys, Arg, Asn, His, Asp, Tyr, and Gln. For BS3, mass shift for cross-linked products set to 138.068 Da, modified residues set to Lys. For EDC, the mass shift for cross-linked products was set to −18.011 Da, modified residues set to Lys, Asp, and Glu. Thermo proprietary raw data (.d) files were converted to.mgf files and then searched against a custom database containing the human APOA1 and LCAT sequences. Putative cross-links identified at 1% false discovery rate were assigned if they were identified in all replicates of the cross-linking experiments with the experimental mass within 5 ppm of the theoretical mass.

### Rigid-body docking analysis using DisVis and HADDOCK

The software tool DisVis (25, 26) was used to screen the cross-link constraint list for compatibility with models describing an interaction between an rHDL disc (fixed chain) and a crystal structure of LCAT (scanning chain). The program identifies cross-links that are likely false positives producing a list of restraints that can be used to guide protein docking analyses. The fixed chain was human APOA1 that had been simulated as an rHDL particle with 160 molecules of POPC and 24 molecules of FC (27). In the.pdb file, the two APOA1 molecules (243 a.a. each) were directly connected between the C-terminus of molecule A and the N-terminus of molecule B to give a continuous chain of amino acids from 1-486. The scanning chain was human LCAT (4XWG) crystallized at 2.7 Å (28). A maximal span distance (between beta carbons) was determined empirically for each cross-linker from studies of bovine serum albumin (Supplemental Fig. S1). We used the average spacing for each cross-linker plus 1 standard deviation (SD) as constraints. The cross-links in Supplemental Table S1 appeared to cluster around two binding sites comprised of helix 4/6 on one side of the opposing helix 5 copies (Site A) and helix 6/4 on the other side (Site B). The cross-links in Supplemental Table S1 that were closest to Site A were evaluated by DisVis separately from those closest to Site B. The detailed results of the DisVis analysis are shown in the Online Docking Analysis Supplement. Cross-links compatible with the two binding sites were used as constraints for docking studies using HADDOCK 2.4 (High Ambiguity Driven protein-protein DOCKing) (29, 30), an algorithm that uses a variety of experimentally derived restraints to constrain docking simulations. We used the APOA1 rHDL particle simulation structure (described above, molecule A) and two copies of the crystal structure of LCAT (molecules B and C). We ran parallel analyses using two crystal structures of LCAT (4XWG or 6MVD). The rHDL.pdb file was modified to list all lipids as HETATMs and so they did not conflict with protein residue and chain names. Additionally, all hydrogens were removed from the structure as this improved computation speeds. We noticed no difference in results when hydrogens were present. Prior to the HADDOCK analysis, the rHDL particle and each LCAT molecule were separately fit into both the flipped and unflipped cryo-EM envelopes (as.mrc files) using PowerFit (26). The resulting centroid coordinates were used as ambiguous restraints for the docking along with the cryo-EM envelopes.mrc file. We also ran two combinations of the rHDL disc called “up” and “down” distinguished the position of the hairpin N-terminal domain on one side of the simulated disc. This resulted in 8 possible docking combinations that needed to be analyzed. The details of the docking analyses and results are laid out in the Online Docking Analysis Supplement.

### Specimen preparation for negative-staining TEM imaging

Specimens were prepared for EM using the optimized negative staining protocol to minimize the rouleaux artifact (31, 32, 33). Briefly, purified HDL-LCAT containing fractions were pooled and concentrated to a final protein concentration of 0.1 mg/ml. Before staining, samples were further diluted in a standard Tris buffer to approximately 0.001 mg/ml. Formvar or parlodion/carbon-coated copper TEM grids were glow discharged in a glow discharge unit (Electron Microscopy Sciences EMS100) at 25 mA current for 2 min at negative polarity. Immediately after glow discharge, a grid was floated on a 20 μl drop of sample for 30 s and excess sample was blotted from the surface. The grid was washed sample-side-down on two 35 μl drops of deionized water and then applied to two 35 μl drops of 0.75% uranyl formate stain (pH ∼4.5) for 1–1.5 min, gently blotting off excess fluid on the grid between each step. The grid was carefully placed in a plastic Petri dish and stored in a dark place to air dry. The grids were imaged on a Thermo Fisher Scientific (Philips/FEI) T-12 operated at 100 kV.

For two-dimensional classification, images captured on the Tecnai 12 were collected at 0 and 15 degrees of tilt at 210,00X magnification (pixel size of 5.14 Å/pixel) on an AMT sCMOS NanoSprint5 camera. Images were collected with both the AMT camera software and with SerialEM (34) set up in low-dose mode and at a defocus target of 1.0 μm. Micrographs were imported into the RELION-3.0 software package (35) for particle picking, extraction, and 2-D classification. Particles were extracted from the micrographs with a box size of 60 pixels then underwent 2-D classification into 100 classes.

Quantification of distances of two LCATs from each other was measured on negative stain preparations imaged at 300,00X magnification (pixel size of 3.65 Å/pixel) using the Tecnai 12 and AMT camera. Images were magnified to a total magnification of 2,400,00X for measurement using the FIJI image processing/analysis software package. Approximately 25 micrographs were examined from each condition. For each condition we identified particles where two LCATs were visible on a single HDL. The distance between the two LCAT particles was measured from the center of mass of one LCAT to the center of mass of the other LCAT. More than 200 measurements were made for each condition, then frequency distribution histograms and statistical data were generated in GraphPad Prism software.

### Cryo-EM single particle imaging

The rHDL samples for cryo-EM studies were prepared via the cholate dialysis method (22) with a molar ratio of 1:53:18 (APOA1:1-palmitoyl-2-oleoyl-sn-glycero-3-phosphocholine (POPC) to 1,2-dipalmitoyl-sn-glycero-3-phosphoglycerol (DPPG)) which produces rHDL particles of approximately 96 Å in diameter. The negatively charged lipids were included to reduce particle aggregation upon freezing. Recombinant full-length APOA1 was purified from *E. coli* (36). After cholate removal with BioBeads SM-2 (Bio-Rad), the rHDLs were separated from the unreacted protein and lipid on an S200 Superdex 200 Increase 10/300 Gl. An adherent stable cell line was used to express LCAT, which was created by transfecting adherent HEK293F cells with pcDNA4-LCAT and selecting with zeocin. The cell media were replaced every 4–5 days and LCAT was purified via Ni-NTA as previously described (37) followed by HiTrap Phenyl HP (Cytiva). The purified protein was concentrated by filtration and dialyzed against 20 mM HEPES pH 7.5, 150 mM NaCl. The LCAT–rHDL complex was prepared by pre-incubating 100 μM LCAT and ∼25 μM rHDL separately at 37°C for 5 min and then 1:04 PM together for 3 min at 37°C. Hundred microlitre of complex was injected onto a Superdex 200 Increase 10/300 Gl pre-equilibrated with 10 mM Tris, 150 mM NaCl, 1 mM EDTA, pH 8. 3.5 μl of the undiluted peak fraction (9.9 ml retention volume) was applied to glow-discharged ultrathin carbon Quantifoil 2/2 200 mesh grids (Electron Microscopy Services). The sample was then vitrified by plunge freezing into liquid ethane using a Vitrobot Mark IV at 4°C and 100% humidity, with a blot time of 4.5 s and force of 1. Two thousand, four hundred eighty-four micrographs at 450,00x magnification were collected on a Thermo Fisher 200 kV Glacios equipped with a Gatan K2 Summit Direct Electron Detector with a nominal pixel size of 0.98 Å in November 2019. Sixty frames were recorded over 6 s for each image using Leginon (38), resulting in a total dose of 46.05 e^−^/Å^2^.

### Cryo-EM single particle analysis

The cryo-EM data set described above was imported with a spherical aberration 2.7 mm and a gain reference flipped along the Y-axis. The images were motion-corrected using MotionCor2 within RELION version 3.1.2, 2x binning (final pixel size of 1.96 Å), and defaults for all other parameters. The contrast transfer function (CTF) was calculated for each image using ctffind-4.1.14 within RELION, 40,000 Å maximum and 2,500 Å minimum defocus value, and defaults for all other parameters. Two thousand two hundred thirteen images were selected using a minimum CTF resolution cutoff of 5 Å. Initial processing was performed in relion-3.1.2 and then particle stacks were moved to RELION version 4.0.0 for further 3D classification, 3D refinement, per-particle CTF correction, and final particle polishing. An initial round of particle picking was performed using the Laplacian function (min. = 100 Å, max. = 250 Å, threshold = 1, upper threshold = 2) on 100 randomly selected micrographs. Eighteen thousand, nine hundred thirty-four particles were selected having no overlap with the edges of the micrographs. These were extracted with a box pixel dimension of 126 (247 Å) and 2D classification was performed with no masking (mask set to 280 Å), tau = 2 with 25 iterations. Two classes resembling lipid disks containing 4,321 particles were used to re-pick 17,942 particles from the same 100 micrographs (threshold = 1.0, min. inter-particle distance of 100 Å and max. stddev noise = 1.0). Re-classification produced two classes (1,193 particles) in which LCAT features were evident and were used to re-pick (threshold = 0.8) and re-classify from the same 100 micrographs. Two (2) classes (4,237 particles) were used to pick (threshold = 0.6, inter-particle distance of 110 Å) from the entire database of 2,213 images. Four hundred fifty-one thousand, nine hundred sixty-nine particles were extracted excluding outlier pixels (±4 sigma) and were subjected to a final round of 2D classification in RELION 3.2.1. Particles from 2D class averages with at least one LCAT visible were collected and moved into RELION 4.0.1 for Ab initio model building, 3D Classification, and final refinements including per-particle CTF refinement and particle polishing. This initial map was imported into cryoSPARC version 4.6.2 for further particle picking, two- and three-dimensional classification, refinements and particle orientation analyses as follows.

Raw movies were imported into CryoSPARC v4.6.2 and patch motion corrected (12 × 12) with dose correction as above. The CTF values were then estimated using the default parameters in Patch CTF. A micrograph denoiser model was trained using 400 epochs and a grey scale normalization factor of 1.5 and used in a subsequent denoising job. The RELION map was then imported into cryoSPARC, and used for template-based picking on denoised micrographs, without the use of CTFs to filter templates, and with a particle diameter of 200 Å. This resulted in 1,425,296 particles over 2,483 micrographs. Outlier picks (based on CTF, drift, ncc score, etc) were removed using the inspect picks resulting in a more clustered particle stack containing 1,102,770 particles. These particles were then extracted at a box size of 168 pix resulting in 920,122 particles. A curate exposure job was completed, and 144 low-quality micrographs were removed by hand. A secondary inspection pick was completed on this new micrograph/pick set, with 597,170 particles being retained for classification. To address previous failed 2D class jobs (memory error), the particle sets tool was used to split this stack into 2 even stacks. 2D classification was then performed on these particles with no circular mask, inner mask diameter of 260 Å and outer of 280 Å, 40 online-EM iterations, 20 full final iterations, 150 classes, batch size per class of 200, and force max over poses/shift set to false. The highest resolution classes containing visible LCAT molecules (27 total) were then used as templates for an additional round of particle picking in order to remove the potential effect of bias from the volume-generated templates. Picking was therefore performed on denoised micrographs using particle diameter 200 Å, and a minimum separation distance of 0.25 diameters, with the maximum number of local maxima set to 5,000. The 1,824,212 particles picked were then subjected to an inspect picks job to remove outliers/bad picks using thresholds ncc score> 0.550, local power between 59 and 86. These particles were then extracted from the micrographs at box size 170 pixels. A final round 2D classification was completed using the parameters most recently described, and 23 classes containing 101,678 particles were used for initial map generation. Two classes were generated using Ab Initio and further refined via hetero-refine for each class. The largest, most well-defined class was retained and placed under an additional round of non-uniform refinement. Due to a rippling in the real space slices map projections, it was assumed junk particles remained, and thus the particles were placed back into 2D classification using 5 final full iterations and 20 online-EM iterations. Forty-four thousand, three hundred ninety-one clean particles were then used to generate a final Ab Initio Model, which was placed under non-uniform refinement with no masking and a window dataset set to false. This initial model was used to generate a static mask in ChimeraX covering the entire molecule. The outputs and the generated static mask were used as inputs for a final non-uniform refinement yielding a 9.8 Å map.

### LCAT activity assay

#### Radioactivity based

The efficiency of LCAT-catalyzed cholesterol esterification on rHDL was measured according to methods previously described (21, 39). The assay was performed on a particle preparation containing POPC lipids and 10 μCi of [^3^H] cholesterol. Particles (356 nM) were incubated with BSA (60 nM) and LCAT (1.5 pM) at 37°C in STB for 30 min. Lipids were extracted and separated using instant thin-layer chromatography silica gel plates (iTLC-SG; Agilent) with a mobile phase of 600:60:1 (v/v) of petroleum ether, ethyl ether, and acetic acid. The FC and CE bands were excised, and [^3^H] cholesterol counts were determined using scintillation counting. The fractional esterification rate was calculated as described (21).

#### Mass based

In some cases, we utilized a mass-based LCAT assay which avoided the issue of running radioactive rHDL particles on chromatography systems. This assay was performed on particles containing POPC and FC of the same ratios as above. Particles (3.56 μM) were incubated with BSA (60 μM) and LCAT (0.89 pM) at 37°C in STB ± 3 mM dithiothreitol (DTT) for 30 min. Hexane extraction (containing internal standard, stigmasterol [Avanti]) was used to stop the LCAT reactions and remove the lipids from the proteins. The FC, CEs, and phospholipids were all separated by Sep-pak (Waters) purification. FC fractions were analyzed on a Shimadzu GC-2010 with an Agilent HP-5 capillary column. The area under the curve for cholesterol and stigmasterol peaks was used to calculate the cholesterol esterification rate when compared to samples lacking LCAT. Each condition was performed in triplicate.

## Results

### Site-directed mutagenesis to identify APOA1 residues important to the LCAT reaction

**Fig. 1:**
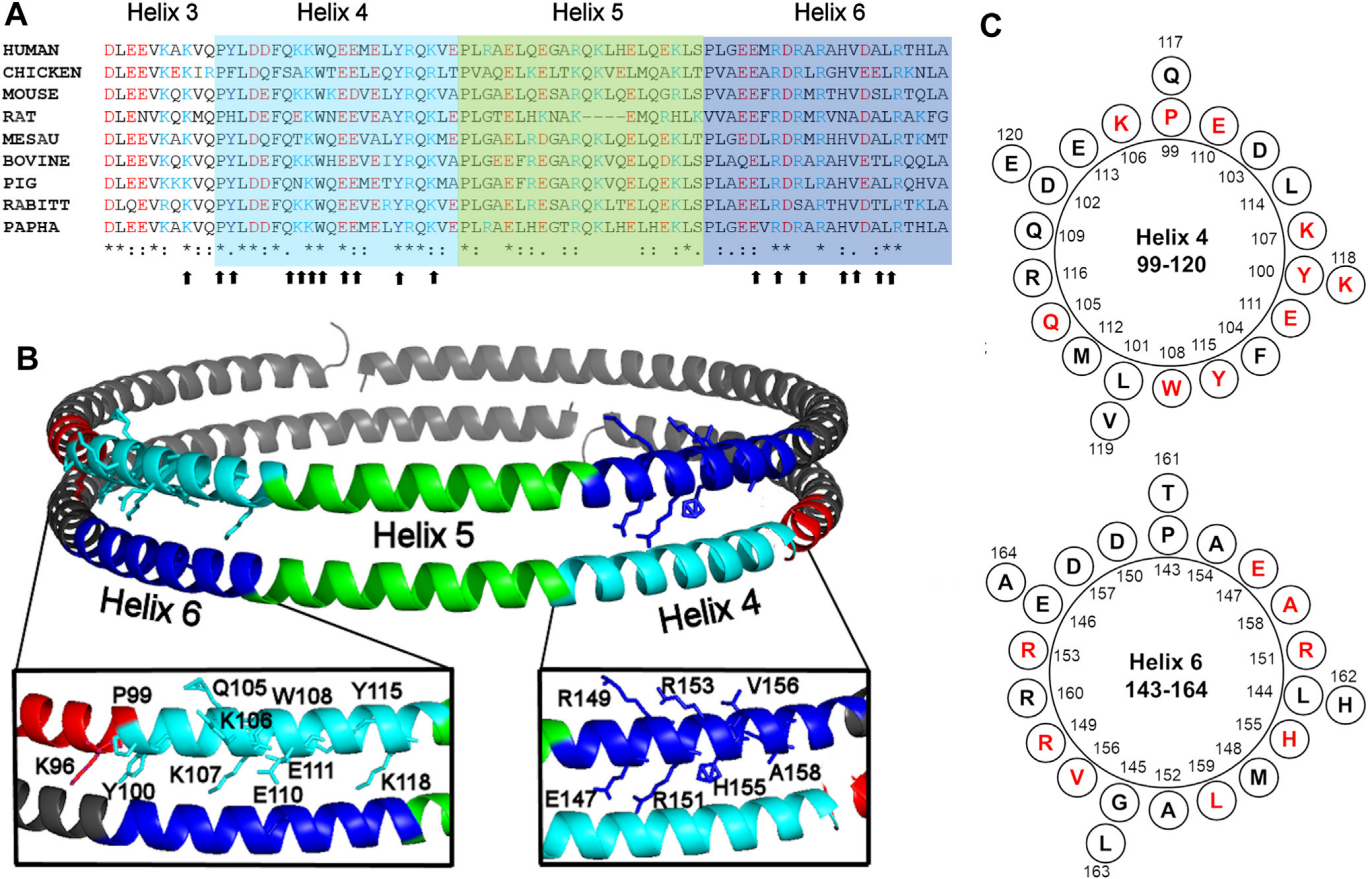
Sequence alignment and location of APOA1 residues targeted for site-directed mutagenesis. A: Mammalian sequence alignment of APOA1 helices 3–6. For all figures in this paper, helix 4 residues (P99-E120) are shaded in cyan, helix 5 residues (P121-S142) are lime green, and helix 6 residues (P143-A164) are dark blue. In the alignment, acidic residues are colored red, basic residues light blue, and neutral or hydrophobic residues in black. Symbols at the bottom of each aligned residue indicate the degree of conservation with an asterisk indicating 100% conservation across these species, two dots indicating one or two conservative substitutions, one dot indicating one non-conservative substitution, and lack of a symbol indicating low conservation. Black arrows indicate residues targeted for mutagenesis (see online Supplemental Table S1 for rationales). B: A cartoon of the theorized double belt model of human APOA1 (18). The helices of interest for this study are colored as above with helix 3 shown in red. Residues targeted for site-directed mutagenesis are shown in ball and stick form. The insets show close-ups of the helix 4/6 and 6/4 interfaces. Images were generated in PyMol (https://pymol.org/). C: Helical wheel projections of helices 4 and 6 showing the mutated residues in red.

Our previous work suggested that residues in helices 4 and 6 of two adjacent APOA1 molecules in HDL form a discontinuous ‘epitope’ for LCAT binding and activation (21, 22). To further test this, point mutagenesis was used to identify specific residues critical to the esterification reaction. We started by performing an APOA1 sequence alignment across multiple mammalian species (BLASTP) (Fig. 1A). In helix 4, 10 residues (P99, L101, D102, F104, K107, W108, E110, Y115, R116 and Q117) are fully conserved. Several residues are charged in the center of the helix (K107, E110, E111) and near the end (K118). Helix 6 also contained highly conserved charged residues E147, R151, and R153. In models of both lipid-free (40) and lipid-bound APOA1 (27), these residues are commonly solvent-exposed and are available to interact with LCAT (Fig. 1B, C). Supplemental Table S1 lists the residues mutated for this study and a brief rationale for each.

**Fig. 2:**
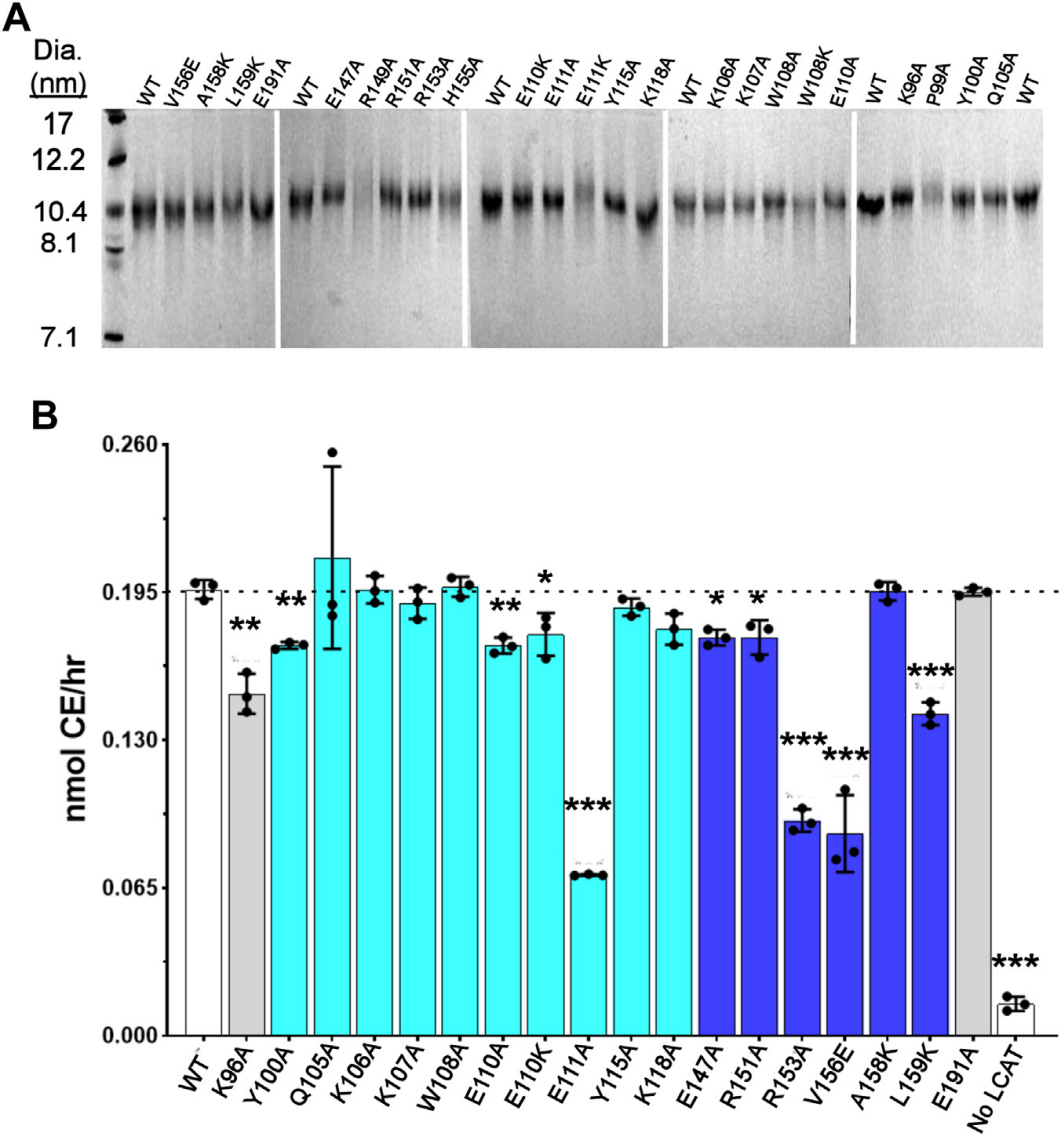
Homogeneity and LCAT reactivity of rHDL particles produced with APOA1 site-directed mutants. A: 8%–25% gradient native PAGGE analysis of rHDL particles generated by cholate dialysis. High molecular weight standards are on the left with hydrodynamic diameters indicated. The WT rHDL particles are included on each gel for direct comparison. Gels were stained with Coomassie blue. B: rHDL particles containing tritiated cholesterol were incubated with human LCAT at an enzyme/substrate ratio that reaches approximately 50% of the apparent V_max_ for rHDL particles with WT APOA1 under these conditions. The incubations ran for 30 min at 37°C. The particles were delipidated by organic solvent extraction and the lipids were separated by thin layer chromatography. Counts in the band corresponding to CE were used to calculate a reaction rate (n = 3). Error bars represent 1 SD. The color of each bar represents the position of the mutated residue in helices 4, 5, and 6 as colored in Figure 1. Experiments were repeated up to three times with independent preparations of many of the particles. A one-way ANOVA with a Holm-Sidak post hoc test was used to determine differences from WT rHDL: ∗*P* < 0.05, ∗∗*P* < 0.005, ∗∗∗*P* < 0.001.

Each APOA1 variant, was expressed, purified (36), and reconstituted into rHDL particles (41) under conditions that generate particles 96 Å in diameter with about 160 molecules of palmitoyl-oleoylphosphatidylcholine (POPC) and 24 molecules of FC. Because LCAT activity is sensitive to rHDL particle diameter (42), we carefully monitored whether the mutations affected the size, composition, or yield of the particles formed. If we saw even minor deviations from the WT protein, we rejected the mutant for further work (see Supplemental Table S1). Figure 2A shows a native PAGGE analysis indicating that most of the point mutants formed rHDL particles with similar hydrodynamic diameter and homogeneity to WT APOA1. Clear exceptions were P99A, W108K, E111K and R149A, which were not analyzed further. All accepted particle preparations exhibited roughly similar phospholipid:protein molar ratios ranging from 80:1 to 90:1.

With included ^3^[H]-FC, the rHDL particles were incubated with human LCAT, and the velocity of CE formation was monitored. We selected substrate concentrations that were ∼50% of the apparent V_max_ so that any differences in reaction rate were not obscured by enzyme saturation. Figure 2B shows that rHDL^WT^ was an effective LCAT substrate producing about 0.2 nmol CE/h. CE production was negligible in the absence of LCAT. Most of the variants exhibited activity comparable to WT, though some exhibited small, but statistically significant, reductions in activity of ∼10% (K96A, Y100A, E110A/K, E147A, and R151A). Other mutants in both helix 4 (E111A) and 6 (R153A, V156E, and L159K) showed significant reductions of 30% to >50%. These results support the notion that *both* APOA1 helices 4 and 6 are important for LCAT-mediated CE formation and these residues likely play key roles at some stage of the reaction cycle.

### Identifying the site(s) of interaction between LCAT and APOA1 in discoidal rHDL by chemical cross-linking

Previous work from the Davidson (21) and Tesmer (22) labs showed that LCAT and APOA1 in rHDL particles can be covalently linked using Lys-specific chemical cross-linking agents. Unfortunately, in human APOA1, there are only 3 Lys residues in helix 4 and none in helix 6, restricting the ability of these reagents to define interaction sites. Therefore, we used three cross-linking agents: BS^3^ (bis(sulfosuccinimidly)suberate), a water-soluble, homobifunctional Lys-to-Lys cross-linker, EDC (1-ethyl-3-(3-dimethylaminopropyl)carbodiimide), a heterobifunctional carboxyl-to-amine linker, and formaldehyde (FM) which can cross-link a number of amino acids, but prefers Arg and Lys residues (24). The rationale was that the combination of possible linkages among these reagents should optimize the chances of finding distance constraints for evaluating the LCAT to APOA1 interaction. To determine the specificity and the distance constraint for each reagent, we tested them with a reference protein of well-defined structure, bovine serum albumin (BSA). Using the cross-link identification criteria laid out in *Methods*, we observed high-confidence cross-links for BS^3^ (14), EDC (16), and FM (26). Supplemental Fig. S1 shows that most cross-links identified within BSA were consistent with a contact plot derived from its crystal structure (3V03) (43) showing spatial proximity of < 24 Å between the α carbons of each residue. However, there were cross-links that spanned up to 50 Å. On average, FM cross-links were most consistent with the structure with an average span of 18 ± 8 Å. BS^3^ was intermediate with 23 ± 10 Å whereas EDC captured longer range connections at 28 ± 15 Å.

**Fig. 3:**
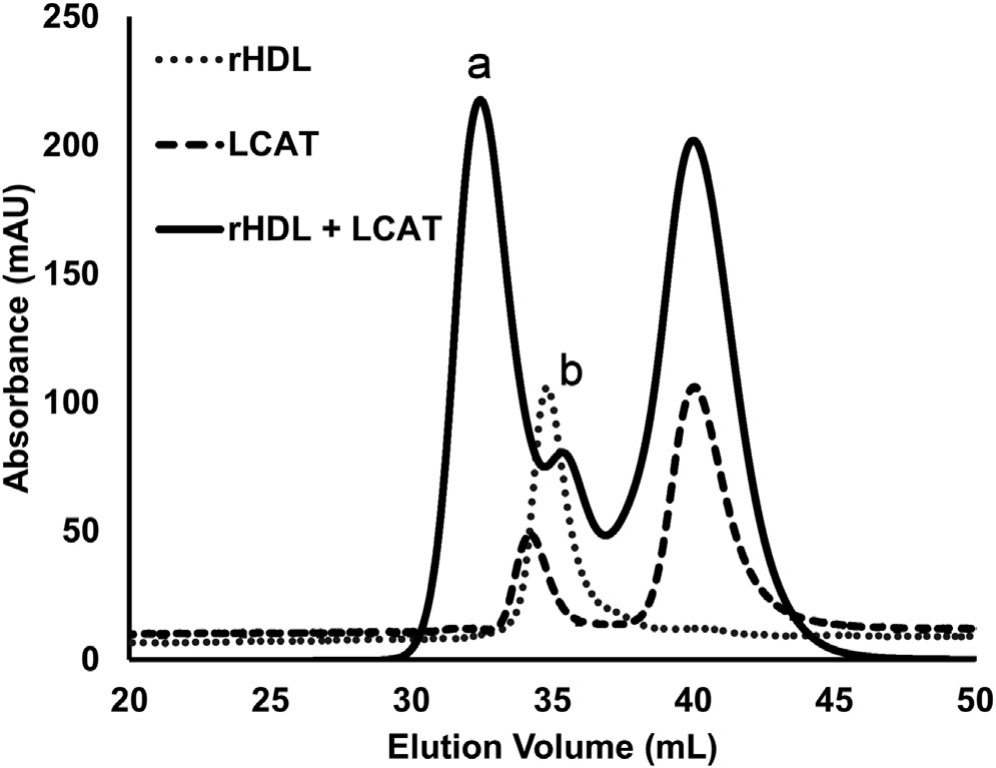
Crosslinking of LCAT to rHDL particles. Size exclusion chromatography of rHDL alone (dotted), LCAT alone (dashed), and rHDL cross-linked with LCAT (solid). Peak a contains complexes of LCAT and APOA1 from the rHDL particles. Peak b reflects the size range of unreacted rHDL particles as well as unreacted dimeric LCAT. The right-most peak contains monomeric LCAT.

Next, rHDL^WT^ particles ∼96 Å in diameter were incubated with human LCAT (3:1 M ratio LCAT to APOA1) for 30 min then cross-linked with all three cross-linking reagents under the conditions described in *Methods*. Using the FM dataset as an example, size exclusion chromatography (Fig. 3) showed the complexes created. The dotted line shows that the rHDL particles alone eluted at about 35 ml as a single peak, demonstrating their homogeneity. The dashed line shows LCAT alone which primarily eluted as a monomer at 42 ml with some dimer at 34 ml. When combined and crosslinked, a significant amount of LCAT remained monomeric at 42 ml. However, about half of the LCAT shifted to a larger complex (peak a) eluting at 31–32 ml, likely covalently linked to either one or two APOA1 molecules. There was a shoulder (peak b) that likely reflected rHDL particles that failed to cross-link with LCAT. Fractions across peak a were exhaustively proteolyzed and subjected to LC-MS/MS analysis. We first evaluated the cross-links involving the APOA1 scaffold on the rHDL particles. Supplemental Fig. S2 shows that all three reagents produced cross-links that were generally consistent with a contact plot derived from the 20 μs simulation of an rHDL-2-100 complex (27). However, there were two general areas of discrepancy. The first involves the N-terminal residue of APOA1 which seems to interact both intra- and inter-molecularly across almost the entire length of APOA1. This has been observed by others (44) and likely reflects high conformational dynamics of the N-terminus. The second region of divergence involved residues between 182-227 interacting with the central helix 5 domain. The nature of these cross-links is not clear but may reflect a population of particles that exhibit an alternate APOA1 helical registry. Nevertheless, most of the cross-links indicated the plausibility of the simulated rHDL model.

We then turned our attention to cross-links between APOA1 and LCAT. Our workflow identified a total of 47 reproducible cross-links across all three reagents (Supplemental Table S2). We excluded cross-links involving the APOA1 N-terminus due to its high dynamics as described above. Lysine 240 in the LCAT lid domain was the most cross-linked residue. The LCAT N-terminus was also highly cross-linked. In general, LCAT crosslinks to APOA1 were clustered in APOA1 helices 4 and 6, with a few in helix 5. Only a small number of linkages were observed outside this domain of APOA1. These 22 cross-links were evaluated as experimental constraints for docking analyses between LCAT and rHDL particles as described below.

### Identifying site(s) of interaction between LCAT and APOA1 in discoidal rHDL by negative stain and cryo-EM

**Fig. 4:**
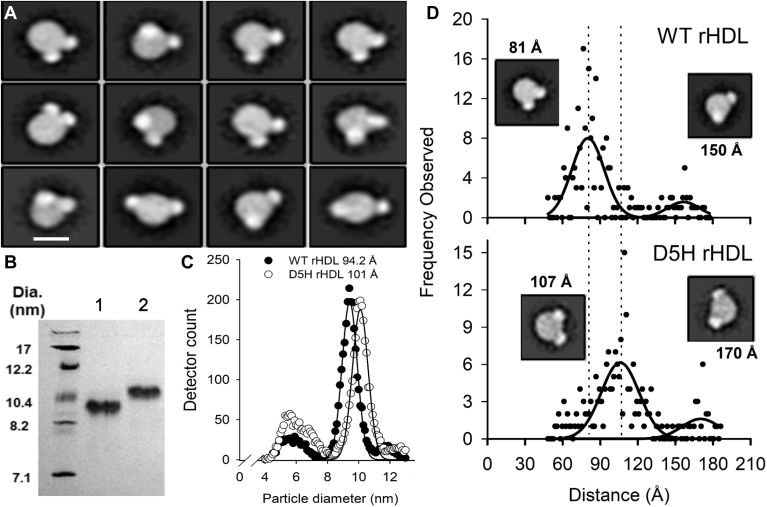
Negative stain EM analyses of LCAT crosslinked to WT and a double fifth helix version of APOA1 (APOA1^D5h^) in rHDL. A: Representative sampling of 2-D class averages obtained with crosslinked LCAT/rHDL^WT^ complexes. The white scale line indicates 10 nm. B: Native PAGGE analysis of rHDL particles generated with WT (lane 1) and APOA1^D5h^ (lane 2). The gel was stained with Coomassie blue. C: Calibrated ion mobility measurements of the same particles. The points show averages of triplicate measurements. D: Image analysis quantitation of the distance between bound LCAT molecules bound to rHDL^WT^ and rHDL^D5h^. Two hundred individual particles were selected at random and the distance between the centers of the two bound LCATs was measured. The data was fit with PeakFit (Systat, San Jose, USA) to determine the average distances. 2-D averages of particles near the apex of each peak are shown as insets.

We previously showed that negative stain EM can visualize LCAT molecules in complex with rHDL (45) and did so again with cross-linked species. Figure 4A shows a series of 2-D class averages for rHDL particles containing WT APOA1 interacting with 2 LCAT molecules. The LCAT molecules mostly interacted at the periphery of the rHDL particles. In most classes, the LCAT molecules appeared close together on one side of the disc. However, fewer classes showed them further apart at various distances depending on the angle of observation.

Because there were no prominent structural features on the APOA1 belt that could act as landmarks to identify the exact location of the observed binding sites, we turned to protein engineering. With the reciprocating structure of the 5/5 double belt (46), additional copies of helix 5 can theoretically be accommodated without perturbing the intermolecular salt bridge interactions predicted to form around the rest of the belt. If LCAT primarily interacts with an epitope comprised of helices 4 and 6 from two different APOA1 molecules then adding another copy of helix 5 should increase the spacing between the two bound LCAT molecules. We generated an APOA1 mutant containing a doubled fifth helix (APOA1^D5h^). Functional analyses demonstrated that APOA1^D5h^ behaved like WT with similar abilities to solubilize lipid liposomes and to promote cholesterol efflux from cells expressing the ATP binding cassette transporter A1 (ABCA1) (Supplemental Fig. S3A, B) (47). Furthermore, APOA1^D5h^ formed homogeneous rHDL particles with POPC. Figure 4B shows a native PAGGE analysis indicating that rHDL^D5h^ exhibited a larger diameter than rHDL^WT^ (103 ± 0.3 vs. 94 ± 0.3 Å, respectively, n = 3). This size increase was confirmed by calibrated ion mobility analysis (cIMA) which measured their diameters at 100 and 94 Å, respectively (Fig. 4C). Composition analysis showed that rHDL^D5h^ contained more lipid than rHDL^WT^ (100 ± 3:1 vs. 86 ± 3:1 M ratio of POPC to APOA1, respectively). Importantly, the addition of the extra helix did not affect the ability of the particles to activate LCAT (Supplemental Fig. S3C).

The distances between the centers of the LCAT molecules bound to both rHDL^WT^ and rHDL^D5h^ were determined by negative stain EM (Fig. 4D). Although the distances between the bound LCATs attached to rHDL^WT^ particles varied somewhat, as seen in Figure 4A, most of the images showed the LCATs to be about 81 Å apart with a second minor population peaking at around 150 Å apart. Thus, ∼85% of the LCAT molecules are bound relatively close together on the WT particle edge. The LCATs bound to rHDL^D5h^ showed a similar bimodal distribution, but both distances increased. Most LCATs shifted to 107 Å apart, a shift of about 26 Å versus those on WT particles. From the cIMA measurements, the circumference of rHDL^WT^ was 300 Å while that for rHDL^D5h^ was 324 Å. Thus, the additional copy of helix 5 increased the rHDL circumference by ∼24 Å (the same calculation for the native PAGGE data yielded a difference of ∼29 Å), consistent with the 26 Å change in LCAT spacing by negative stain EM. This strongly argues that the LCAT molecules bind at the helix 6/4 and 4/6 interface because that is geometrically the only place where their centers of mass can be 81 Å apart, but move 26 Å further with an additional copy of helix 5.

**Fig. 5:**
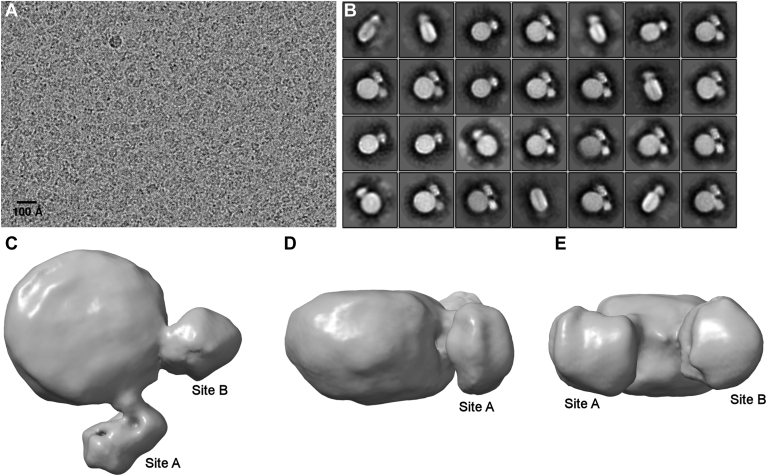
Cryo-EM analyses of LCAT complexed to WT APOA1 rHDL. A: Representative micrograph of the WT APOA1/LCAT complexes. B: selected 2-D class averages of rHDL particles containing bound LCAT molecules. C: “Top” view of a 3-D construction of the complex at approximately 9 Å resolution. D: “Side” view of the complex rotated by 90° around the horizontal axis from panel c, e) View of the complex rotated by 90° around the vertical axis from panel d. Map images were produced in ChimeraX version 1.4 (University of California, San Francisco).

We made multiple attempts to derive high-resolution structural models of an rHDL^WT^ particle with two bound LCAT molecules by cryo-EM. (Fig. 5A). Figure 5B shows selected 2-D class averages of particles with LCAT molecules bound. Again, the LCAT molecules bound close together on the particle edges consistent with the negative stain EM. The improvement in resolution versus negative stain EM was apparent with possible secondary structure features visible in some of the 2-D class averages. There was no evidence of the small population of particles with LCATs binding further apart as seen in the negative stain experiments, which may reflect compression of the particles that can occur with negative staining. A 3-D molecular envelope was derived (Fig. 5C, D, E) with an estimated resolution of 9.8 Å (Supplemental Fig. S4A). Higher resolution envelopes have thus far been stymied by *i*) the relatively low number of rHDL particles that host two LCAT molecules, even when the LCAT to APOA1 molar ratio is high, *ii*) inherent microheterogeneity among the rHDL particles themselves, ie, slight variations in lipid content and angular separation of the two LCAT molecules that complicate class averaging, and *iii*) preferred orientation of complexed particles during freezing (Supplemental Fig. S4B). Even at this resolution, several new pieces of information were apparent. First, the orientation of the LCAT molecules clearly differed at the two binding sites. Second, regardless of orientation, the LCAT molecules contacted the APOA1 belt at specific points with most of the molecule isolated by an aqueous gap.

### Modeling the LCAT APOA1 interaction in simulated rHDL particles

**Fig. 6:**
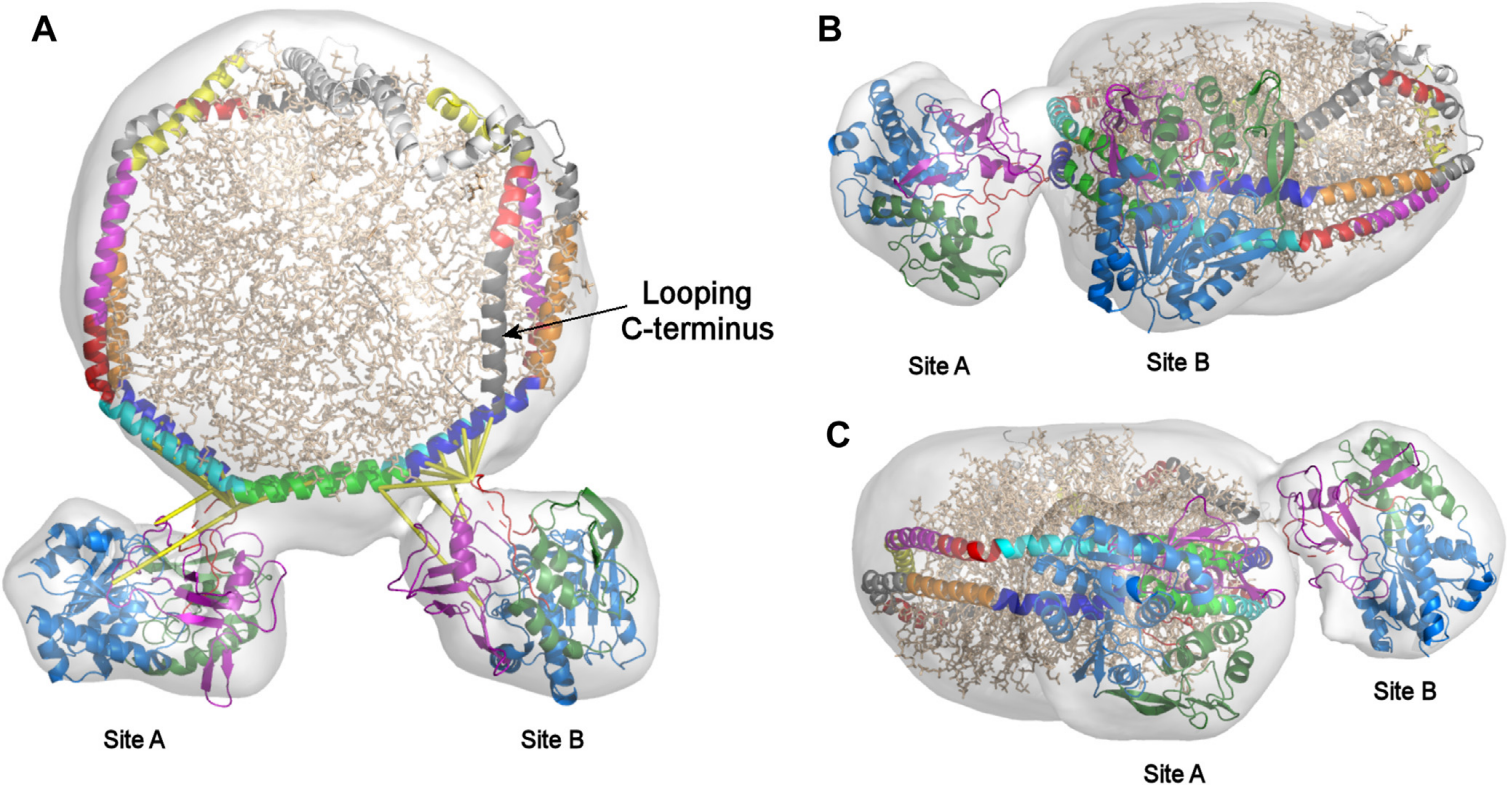
Best-fit model of a simulated 100 Å rHDL particle binding two LCATs using chemical cross-links and the cryo-EM envelop as restraints. A 20 μs molecular dynamics simulation of the rHDL-2-100 particle (27) was docked with the crystal structure of human LCAT (4XWG) as described in *Methods* and the *Online Docking Supplement*. A: Top view showing the APOA1 helices colored as H1, yellow; H2, pink; H3, red; H4, cyan; H5, green; H6, blue; H7, orange; H8, light gray; H9, red; H10, dark gray. POPC molecules in the center are shown in ball-and-stick in tan. LCATs docked to sites A and B are indicated. The LCATs are colored with the α/β hydrolase domain (residues 21–31, 120–213) in blue, the membrane binding domain (MBD, residues 32–119) in purple, and the cap domain (residues 214–303, 319–344) in forest green. The lid region (residues 226–249) is shown in red for both LCATs. The cryo-EM envelope is shown in transparent gray. Chemical cross-links identified as relevant to the interaction by DisVis and HADDOCK are shown with yellow lines. B: Detail of “parallel” LCAT interacting with binding site A. C: Detail of “perpendicular” LCAT interacting at binding site B. All images generated in PyMol version 2.5.5 (Schrodinger, LLC).

The cryo-EM data was not of sufficient resolution to generate a detailed model for the APOA1/LCAT interaction so we pursued an integrated molecular docking strategy using the HADDOCK platform (48). We began with high-resolution X-ray crystal structures of human LCAT in two different conformational states (28, 45) and our molecular dynamics simulation model for a 100 Å rHDL particle (27), which was strongly validated by the cross-linking data obtained here (Supplemental Fig. S3). The docking of LCAT to the rHDL was guided using experimentally derived constraints from chemical cross-linking and the cryo-EM density map. A detailed description of the docking analyses is provided in the Online Docking Supplement. Briefly, the list of cross-links was screened for false positives using DisVis, indicating the likelihood of two LCAT binding sites at either end of helix 5. Because molecular handedness could not be determined from the cryo-EM map, we performed the docking analysis with both unflipped and flipped versions of the map. Additionally, we tested both an “up” and “down” orientation of the rHDL particle with respect to the two LCAT binding sites. The best docking results, as measured by the lowest HADDOCK score, lowest restraint violation energies, and overall shortest cross-linker lengths, resulted from the flipped EM map, the 4XWG LCAT structure with the rHDL particle in the “up” orientation (Fig. 6). The 6MVD (or lid ‘open’ structure of LCAT) resulted in higher restraint violation energies and significantly longer cross-linker spans versus the lid ‘closed’ 4XWG structure. As implied by the cryo-EM data, the LCATs occupying the two binding sites on APOA1 were in distinct orientations, one in which the long axis of the LCAT molecule lay parallel to the APOA1 belt helices (Site A) with the other rotated about 90° in a perpendicular orientation to the helical belts (Site B). The LCAT structures fit well within the cryo-EM maps at both binding sites. The LCAT at site A interacted with both helices 6 and 4 of APOA1 via both its cap domain (residues 214–303, 319–344) and its membrane binding domain (MBD, residues. 32–119). As mentioned above, Lys240 at the distal tip of a flexible loop in the cap domain was the most active residue in intermolecular contacts with APOA1 by cross-linking. In fact, we found about an equal number of crosslinks between this residue and those in both helix 6 and 4 of APOA1. LCAT residue 240 also strongly interacted with APOA1 helix 6 at site B, but more distal to the helix five-sixths junction than in site A. The LCAT α/β hydrolase domain (in blue in Fig. 6) was situated away from the disc edge across an aqueous gap and we found no crosslinks between this domain and APOA1. The same LCAT domains faced the disc edge at site B, but they were shifted in orientation versus site A. The molecule was in closer proximity to APOA1 helix 6 and the MBD was situated higher on the disc edge, possibly positioned for interactions with the phospholipid headgroups.

### The role of APOA1 helical proximity in LCAT activation

**Fig. 7:**
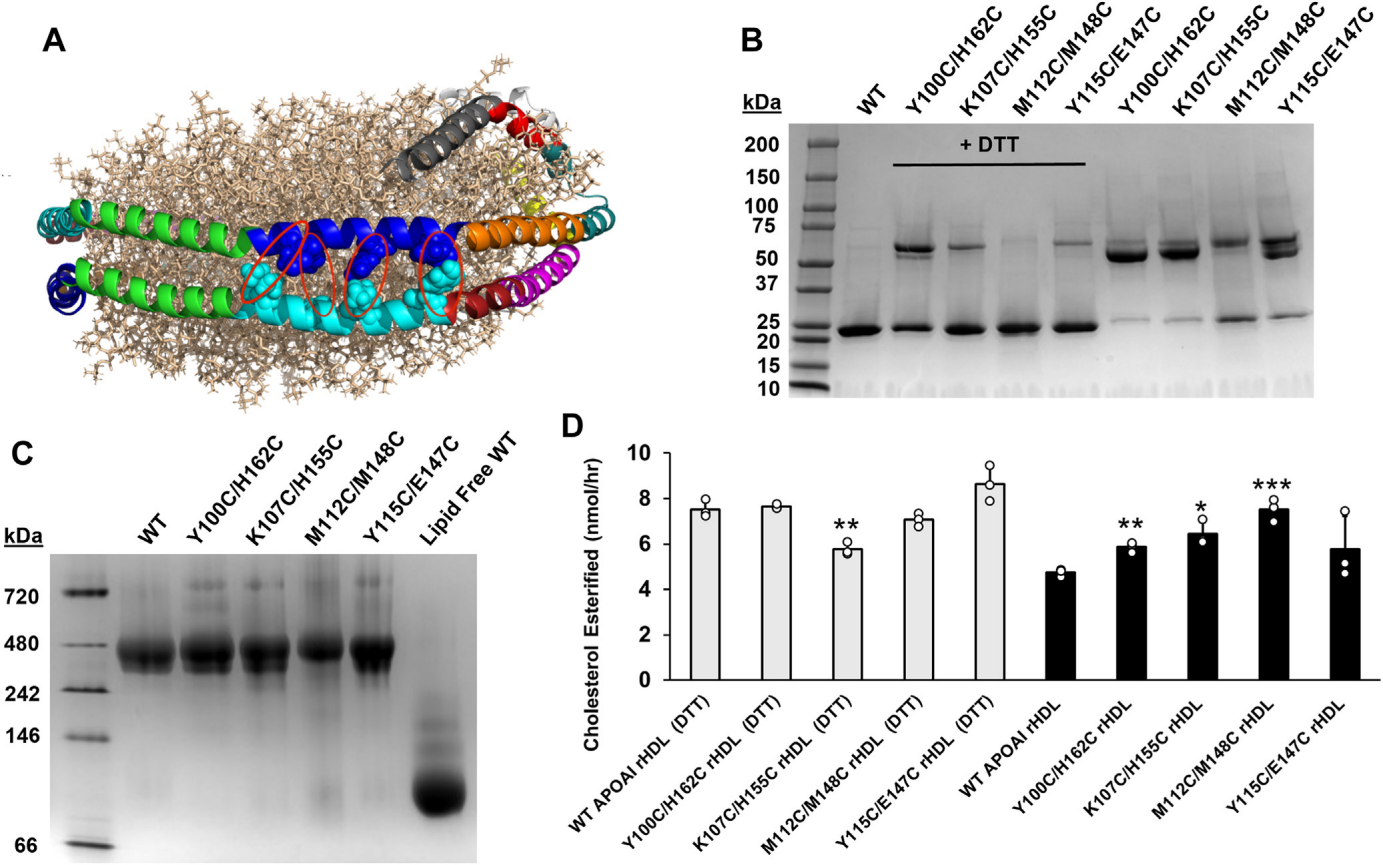
Effect of the covalent linkage of APOA1 helices 4 and 6 on LCAT activity. A: Cys mutations were placed in APOA1 to lock helices together based on the simulated rHDL-2-100 particle. Helices 4 and 6 are shown with the same color scheme as Figure 1. Red ovals show anticipated disulfide linkages between strategically introduced Cys residues (indicated in spacefill). B: SDS-PAGE analysis of rHDL particles generated with the double Cys mutants under reducing conditions (right) and oxidizing conditions (left). Monomeric APOA1 is 28 kDa and dimeric is ∼56 kDa. C: Native PAGGE analysis of rHDL particles generated with double Cys mutants. All gels were stained with Coomassie blue. D: LCAT activity under reducing conditions (minimal disulfide linkages) in white and under oxidizing conditions (maximal disulfide linkages) in black. Error bars represent 1 SD. Each condition was analyzed by a non-radioactivity-based LCAT activity assay (n = 3). Two-tailed *t* test between each double Cys mutant and its corresponding WT control ∗*P* < 0.05 ∗∗*P* < 0.005, ∗∗∗*P* < 0.001.

The docking model described above, combined with our previous results indicating that APOA1 helices 6 and 4 were required to be in registry for effective LCAT activation (21), suggested that LCAT might access the HDL particle core via a portal created by helices 6 and 4 moving apart during the enzymatic reaction cycle. For example, the hydrophobic product CEs could be effectively transferred from LCAT, between APOA1 helices 4 and 6, into the hydrophobic acyl chain regions of the disc lipid bilayer. To test this idea, we designed double Cys mutants of APOA1 to produce intermolecular disulfide linkages between helices 6 and 4 when APOA1 is in the double belt configuration on an rHDL particle. These residues were placed directly across the APOA1 molecular interface along the length of the helical pair (Fig. 7A). Y100C/M162C links the N-terminal end of helix 4 to the C-terminal end of helix 6. K107C/H155C links the two helices at their approximate centers, while M112C/M148C and Y115C/E147C link the C-terminal end of helix 4 to the N-terminal end of helix 6. These latter two mutants are close to the LCAT to APOA1 binding site as modeled for both sites A and B. Our idea was to covalently ‘sew’ the two helices together so they could not separate during the LCAT reaction. Figure 7C shows that all mutants were capable of generating 96 Å rHDL particles matching those generated with WT APOA1 by native PAGGE. Even though the particles were generated under reducing conditions, we noticed some covalent dimer formation by SDS PAGGE (Fig. 7B), particularly for Y107C/H155C and Y100C/H162C. However, when the reducing agent was removed, most of the mutants dimerized extensively with only about 10% of the APOA1 remaining monomeric. M112C/M148C dimerized less efficiently but still formed about 50% dimer under oxidizing conditions. We measured the ability of LCAT to esterify cholesterol in a mass-based assay (see *Methods*). When the disulfide bonds between APOA1 molecules were present, LCAT showed a similar (or in some cases slightly better) ability to esterify cholesterol than the particles containing WT APOA1 (Fig. 7D). Under reducing conditions ie, disulfide bonds are largely absent, LCAT activity was only marginally affected in some of the mutants. This indicates that the Cys substitutions themselves, irrespective of the disulfide bonds, did not significantly perturb the LCAT function. Overall, these data strongly suggest that APOA1 helices 4 and 6 can remain in tight proximity throughout the LCAT reaction cycle.

## Discussion

The nature of LCAT interaction with APOA1 in HDL has been debated for decades. Early LCAT models showed that the domain between Cys 50–74 resembled a loop on pancreatic lipase that forms part of its lipid binding interface with micellar substrates (49), implying LCAT bound to the lipid component of HDL. However, more recent evidence has implicated the central helical domain of APOA1, particularly residues 143–186, in LCAT/HDL interactions (reviewed in (20, 50, 51)). The current study unequivocally shows: *i*) LCAT binds to preferred domains on the APOA1 belt at the helix 6/4 and 4/6 interfaces (sites A and B, respectively), *ii*) specific residues in both helices 6 and 4 impact LCAT activity when mutated, *iii*) the LCATs bound at sites A and B are in orthogonal orientations with respect to the plane of the disc bilayer and, *iv*) the helix 6/4 interface is not disrupted during the LCAT reaction cycle. These observations are discussed with respect to the stimulation of LCAT activity by APOA1 below.

### Reciprocal LCAT binding sites on APOA1?

LCAT interacts with nascent rHDL particles in at least two reciprocal discontinuous binding sites created by the intermolecular interface of helices 4 and 6 of APOA1. The negative stain data indicated the possibility of additional binding locations of much lower occupancy, but no evidence of this was apparent in the cryoEM data. We therefore suggest that the helix 4/6 interface likely reflects the highest affinity LCAT interaction sites in APOA1.

In the rHDL particle simulation structure, the helix 4/6 and 6/4 sites are mirror images of each other with respect to helix 5. Therefore, we found it surprising that the LCAT molecules bound each in orthogonal orientations. One possibility is that the APOA1 helical belt zigzags across the disc edge such that the helix 4/6 and 6/4 pairs (and their associated LCAT molecules) adopt different orientations with respect to the particle face. Direct visualization of the APOA1 belts is not yet possible in our hands, but such undulations seem unlikely as it would require severe twisting in this region that we have never observed in molecular dynamics simulations of these particles (27, 52). It is also possible that the binding of LCAT to one site causes a conformational change in APOA1 that affects LCAT orientation at the second site. Finally, the C-terminus of APOA1 might alter LCAT binding. In particles of this size and composition, our simulations consistently show that the C-terminus of one of the APOA1 molecules loops on itself and traverses back along the particle edge (27) reaching all the way to helix 6 (see arrow in Fig. 6). The C-terminus of the other APOA1 molecule simply continues around the disc to close the belt. This looping helix might alter either lipid packing or the conformation of the proximal 6/4 helical interface to modify LCAT binding at that one site. Such a regulatory role for the APOA1 C-terminus is intriguing because simulations of smaller 80 Å rHDL show the C-termini of *both* APOA1 molecules looping back (8). LCAT activity and binding on these smaller particles is heavily curtailed (21). However, larger particles of 110 Å lack looping termini altogether (27) and are highly efficient LCAT substrates (42). Tests of these ideas are underway with different-sized rHDL particles and those lacking the C-terminal helix.

### Implications for the mechanism of APOA1 activation of LCAT

LCAT structure is dominated by a eight stranded β-sheet stabilized on each side by α-helices within its α/β hydrolase domain (37) which contains the catalytic triad and most of the active site pocket. Two other domains include the membrane binding domain (MBD, residues 36–101) and a cap domain containing a flexible lid (residues 226–249) (37) (see cartoon Supplemental Fig. S5). Manthei *et al.* proposed a mechanism whereby the LCAT lid, normally covering the LCAT active site, swings to an ‘open’ conformation (45). The opened loop then mediates interactions with APOA1 and facilitates lipid movement into the active site (53). The MBD, as its name implies, is critical for lipid interactions as is a highly flexible sequence at the LCAT extreme N-terminus. The MBD is also the primary binding site for piperidinylpyrazolopyridine activators of LCAT (45). In our docking analyses, we tested LCAT crystal structures in which the lid domain is in the “closed” position (PDB entry 4XWG) and the “open” position maintained by one such small molecule activator (PDB entry 6MVD) (45). The best scoring docked structures derived from 4XWG. In 6MVD, the open lid is swung away from the APOA1 belts but comes into close contact with APOA1 helix 6 when closed in 4XWG with substantially shorter average cross-linker spacing distances, particularly at site A. That the lid plays a direct role in APOA1 interaction is consistent with our prior observations that lid residue I233 is important for HDL binding (54). Additionally, at site A, the MBD is oriented directly across from the APOA1 belts and occupies part of the EM density that bridges LCAT and APOA1, supporting its role in a protein-protein interaction. This is consistent with our previous studies showing that mutations at MBD residues W48 and L70 impact LCAT binding to HDL (54). Also of note, the piperidinylpyrazolopyridine small molecule LCAT activator binding site is situated in the middle of the LCAT-HDL interface in both binding poses. In putative site B, the lid is slid upward and primarily interacts with helix 6 while the MBD is shifted away from the belt and toward the surface of the phospholipid bilayer. This positioning of the MBD could be consistent with ‘head first’ extraction of substrate polar lipids like cholesterol and phospholipid from the bilayer to slide into the LCAT active site pocket which faces the APOA1 belts (Supplemental Fig. S5). Binding site B also puts LCAT near APOA1 residues (E111, R153, and V156) whose mutations affect LCAT activity in Figure 1. Interestingly, these residues are relatively distant from the LCAT interaction at site A. Another important lipid binding element in LCAT is its N-terminal 20 amino acids which adopt a highly dynamic structure that has not yet been visualized by X-ray diffraction (28) and thus not included in our docking models. At the risk of pushing our cryo-EM data too far, we generated a full-length human LCAT model using AlphaFold 3 that matched well with the LCAT crystal structures but rendered the N-terminal 20 and the C-terminal 32 residues (also missing from the crystal structures) as low confidence extended structures. We then fit this into the cryoEM map adjusting the N- and C-terminal regions to fit into two unfilled regions of electron density (Supplemental Fig. S6). The model suggests that the LCAT N-terminus traverses across the α/β hydrolase domain putting the N-terminus close to the point where LCAT interacts with APOA1. This is consistent with several high-confidence cross-links found between LCAT residue 1 and the APOA1 4/6 helical interface, explaining the strong effect the N-terminus has on LCAT binding to HDL. The LCAT C-terminus also traverses back across the α/β hydrolase domain but on the opposite side of the molecule (facing away from the rHDL), nicely filling empty electron density in the map. Confirmation of this full-length LCAT model awaits higher-resolution data. Finally, the mechanistic implications of the two distinct binding poses for LCAT on HDL are not yet clear. It could be that the two orientations reflect different steps of the complex LCAT reaction or one may result due to the high ratios of LCAT to rHDL particle that we used for these studies. See Supplemental Fig. S5 for more detailed speculation.

The residues in APOA1 helices 4 and 6 are highly conserved across species, contrasting to the relative lack of conservation just a few positions away in helix 5 (Fig. 1). Since APOA1 helices 4 and 6 need to be in direct registry for full LCAT activity (21), we speculated that APOA1 might enhance LCAT activity by allowing access to the hydrophobic core of the particle, perhaps to deposit the hydrophobic CE product without the requirement to push it through an unfavorable aqueous barrier. The most straightforward way to do this is to form a channel or gap between the two helices at some point in the LCAT reaction cycle. However, our dual Cys mutants clearly show that LCAT works perfectly well even when APOA1 helices 4/6 are covalently sewn together. This indicates that, while LCAT must bind to the APOA1 helical 6/4 pair, it obtains its substrates from the HDL lipid surfaces. It follows that it also deposits product CE through the lipid surface. Rather than forming a channel, the helix 6/4 epitope of APOA1 could concentrate substrate cholesterol molecules near where LCAT interacts. Certain proteins that bind cholesterol contain specific sequences called cholesterol recognition/interaction amino acid consensus sequences (CRAC) domains (55). Dergunov (56) showed that APOA1 contains 5 CRAC domains, 3 located in the N- and C-termini regions. Interestingly, the other two are in APOA1 helix 4 (V97-K106) and in helix 6 (L163-R171), aligning directly across the molecular interface in the 5/5 double belt. The importance of these sites in facilitating LCAT function remains to be explored.

Our data suggests that bihelical peptide constructs designed to mimic the helix 4/6 interface might be a promising approach for enhancing LCAT activity in individuals with partial LCAT deficiency or to test the idea that enhanced LCAT function in normal individuals could be atheroprotective. Moreover, our results hint that other LCAT-activating proteins, like APOE, may have a similar reciprocating binding motif. APOE is thought to be an important activator of LCAT in the cerebral spinal fluid (57) and the interaction may be pivotal for esterifying neurotoxic hydroxysterols that accumulate in the brain (58). Identification of a common mechanism for apolipoprotein activation of LCAT could open the door to treatment strategies in the brain.

Finally, we suggest that the integrative modeling approach taken here (ie, using combinations of lower-resolution structural, functional, and computational techniques to generate more detailed models) will become a standard approach for tackling the challenges of lipoprotein structural biology. The inherent microheterogeneity of lipoproteins, even when reconstituted under controlled conditions in the laboratory, has thus far precluded the generation of atomic resolution data for lipoproteins by cryo-EM. However, lower-resolution electron density maps become powerful guides when used in combination with other techniques such as chemical cross-linking. Indeed, recent cryo-EM structures of apolipoprotein B in human low-density lipoprotein have been of limited resolution, but supplementation with cross-linking and computational techniques has led to exciting new insights into how these particles bind their receptor (59, 60).

## Data availability

All data used to produce this report is available by contacting the corresponding author. The docked structure of the rHDL particle and two LCAT molecules can be accessed in the Protein Data Bank under ID: 9MXZ.

## Supplemental data

This article contains supplemental data (24, 25, 26, 27, 28, 29, 30, 54, 61, 62, 63, 64).

## Conflict of interest

The authors declare that they have no conflicts of interest with the contents of this article.

## Supplementary data

LCAT_interaction_JLR_Rev_2

LCAT Manuscript Data Supplement New
