# Supplementary material for: Lecithin:cholesterol acyltransferase binds a discontinuous binding site on adjacent apolipoprotein A-I belts in HDL
Source: J Lipid Res. 2025 Mar 25;66(5):100786. doi: 10.1016/j.jlr.2025.100786 (PMC12049944; doi:10.1016/j.jlr.2025.100786)
Supplement: LCAT_interaction_JLR_Rev_2 [file mmc1.docx]

**Lecithin:Cholesterol Acyltransferase Interacts With a Discontinuous Binding Site on Adjacent Apolipoprotein A-I Belts in High Density Lipoproteins**

Bethany Coleman^1^, Shimpi Bedi^2^, John H. Hill^3^, Jamie Morris^2^, Kelly A. Manthei^4^, Rachel C. Hart^5^, Yi He^6^, Amy S. Shah^7^, W. Gray Jerome^5^, Tomas Vaisar^6^, Karin E. Bornfeldt^6^, Hyun Song^8^, Jere P. Segrest^8^, Jay W. Heinecke^6^, Stephen G. Aller^3^, John J. G. Tesmer^9^ and W. Sean Davidson^2*^

^1^Department of Molecular and Cellular Biosciences, University of Cincinnati, Cincinnati OH 45237

^2^Department of Pathology and Laboratory Medicine, University of Cincinnati, Cincinnati OH 45237

^3^ Department of Pharmacology and Toxicology, University of Alabama at Birmingham, Birmingham, Alabama 35294

^4^ Life Sciences Institute, University of Michigan, Ann Arbor, Michigan 48109

^5^ Department of Pathology, Microbiology and Immunology, Vanderbilt University School of Medicine, Nashville, Tennessee 37232

^6^ Department of Medicine, University of Washington School of Medicine, Seattle, Washington 98109

^7^ Department of Pediatrics, Cincinnati Children’s Hospital Medical Center and the University of Cincinnati, Cincinnati, OH 45229

^8^ Department of Medicine, Vanderbilt University Medical Center, Nashville, Tennessee 37232

^9^ Departments of Biological Sciences and Medicinal Chemistry and Molecular Pharmacology, Purdue University, West Lafayette, IN, 47907

**Molecular Docking between APOA1 in rHDL and LCAT using DISVIS and HADDOCK**

**Cross-linking strategies:** We performed multiple crosslinking experiments using three complementary cross-linking agents: BS^3^ (bis(sulfosuccinimidly)suberate), a water soluble, homobifunctional Lys-to-Lys cross-linker, EDC (1-ethyl-3-(3-dimethylaminopropyl)carbodiimide), a heterobifunctional carboxyl-to-primary amine linker, and formaldehyde (FM) which can cross-link a number of amino acids, but prefers Arg and Lys residues ^1^. We reasoned that the combination of possible linkages among these reagents would optimize the chances of finding distance constraints for evaluating the LCAT to APOA1 interaction. See *Methods* for details.

**Selection of highest confidence cross-links:** Our workflow outlined in *Methods* identified 47 cross-links across all three reagents at a 1% false discovery rate. Identifications were accepted if they were identified in all replicates in our experiments and were within 5 ppm of the theoretical mass. These are listed in **Table S1**.

**Table S1. Chemical cross-links between APOA1-containing rHDL and human LCAT used for molecular docking analyses.**

|  | **Residue^a^** | |  | **DisVis notation** | |  |  | |  | | |  | |
| --- | --- | --- | --- | --- | --- | --- | --- | --- | --- | --- | --- | --- | --- |
| **#** | **APOA1** | **LCAT** |  | **APOA1 molecule A^b^** | **APOA1 molecule B** |  | **Linker** | **Forms^c^** | | **Hits** | | | **ppm^d^** |
| 1 | R116 | K240 |  | A 116 CB A 240 CB 0 26.7 | A 359 CB A 240 CB 0 24 |  | FM | | 2 | | 71 | | -0.35 |
| 2 | R123 | K240 |  | A 123 CB A 240 CB 0 26.7 | A 366 CB A 240 CB 0 24 |  | FM | | 1 | | 35 | | -0.26 |
| 3 | R131 | K240 |  | A 131 CB A 240 CB 0 26.7 | A 374 CB A 240 CB 0 24 |  | FM | | 1 | | 76 | | -0.67 |
| 4 | R149 | K240 |  | A 149 CB A 240 CB 0 26.7 | A 392 CB A 240 CB 0 24 |  | FM | | 2 | | 17 | | -0.61 |
| 5 | R151 | K240 |  | A 151 CB A 240 CB 0 26.7 | A 394 CB A 240 CB 0 24 |  | FM | | 1 | | 90 | | -0.61 |
| 6 | R153 | K240 |  | A 153 CB A 240 CB 0 26.7 | A 396 CB A 240 CB 0 24 |  | FM | | 1 | | 54 | | -0.76 |
| 7 | R171 | K240 |  | A 171 CB A 240 CB 0 26.7 | A 414 CB A 240 CB 0 24 |  | FM | | 1 | | 89 | | -0.04 |
| 8 | R173 | K240 |  | A 173 CB A 240 CB 0 26.7 | A 416 CB A 240 CB 0 26.7 |  | FM | | 1 | | 26 | | -0.82 |
| 9 | K106 | F1 |  | A 106 CB A 1 CB 0 33.2 | A 349 CB A 1 CB 0 33.2 |  | BS^3^ | | 1 | | 32 | | 0.51 |
| 10 | K106 | K240 |  | A 106 CB A 240 CB 0 33.2 | A 349 CB A 240 CB 0 33.2 |  | BS^3^ | | 1 | | 12 | | -0.61 |
| 11 | K107 | F1 |  | A 107 CB A 1 CB 0 33.2 | A 350 CB A 1 CB 0 33.2 |  | BS^3^ | | 2 | | 131 | | -0.05 |
| 12 | K107 | K240 |  | A 107 CB A 240 CB 0 33.2 | A 350 CB A 240 CB 0 33.2 |  | BS^3^ | | 1 | | 21 | | 0.28 |
| 13 | K118 | F1 |  | A 118 CB A 1 CB 0 33.2 | A 361 CB A 1 CB 0 33.2 |  | BS^3^ | | 1 | | 179 | | -0.07 |
| 14 | K118 | K116 |  | A 118 CB A 116 CB 0 33.2 | A 361 CB A 116 CB 0 33.2 |  | BS^3^ | | 1 | | 36 | | -0.73 |
| 15 | K118 | K240 |  | A 118 CB A 240 CB 0 33.2 | A 361 CB A 240 CB 0 33.2 |  | BS^3^ | | 1 | | 33 | | -0.29 |
| 16 | K133 | F1 |  | A 133 CB A 1 CB 0 33.2 | A 376 CB A 1 CB 0 33.2 |  | BS^3^ | | 1 | | 221 | | -0.08 |
| 17 | K133 | K240 |  | A 133 CB A 240 CB 0 33.2 | A 376 CB A 240 CB 0 33.2 |  | BS^3^ | | 1 | | 23 | | -0.98 |
| 18 | K140 | F1 |  | A 140 CB A 1 CB 0 33.2 | A 383 CB A 1 CB 0 33.2 |  | BS^3^ | | 2 | | 348 | | -0.70 |
| 19 | K140 | K240 |  | A 140 CB A 240 CB 0 33.2 | A 383 CB A 240 CB 0 33.2 |  | BS^3^ | | 1 | | 17 | | -0.89 |
| 20 | K182 | F1 |  | A 182 CB A 1 CB 0 33.2 | A 425 CB A 1 CB 0 33.2 |  | BS^3^ | | 1 | | 121 | | 0.12 |
| 21 | K195 | F1 |  | A 195 CB A 1 CB 0 33.2 | A 438 CB A 1 CB 0 33.2 |  | BS^3^ | | 1 | | 61 | | 0.01 |
| 22 | K206 | F1 |  | A 206 CB A 1 CB 0 33.2 | A 449 CB A 1 CB 0 33.2 |  | BS^3^ | | 1 | | 65 | | 0.28 |
| 23 | K208 | F1 |  | A 208 CB A 1 CB 0 33.2 | A 451 CB A 1 CB 0 33.2 |  | BS^3^ | | 1 | | 63 | | 0.17 |
| 24 | K88 | F1 |  | A 88 CB A 1 CB 0 33.2 | A 331 CB A 1 CB 0 33.2 |  | BS^3^ | | 2 | | 57 | | 0.35 |
| 25 | K94 | F1 |  | A 94 CB A 1 CB 0 33.2 | A 337 CB A 1 CB 0 33.2 |  | BS^3^ | | 1 | | 597 | | 0.32 |
| 26 | K96 | F1 |  | A 96 CB A 1 CB 0 33.2 | A 339 CB A 1 CB 0 33.2 |  | BS^3^ | | 1 | | 313 | | 0.22 |
| 27 | E111 | F1 |  | A 111 CB A 1 CB 0 43 | A 356 CB A 1 CB 0 43 |  | EDC | | 1 | | 48 | | -0.01 |
| 28 | E125 | F1 |  | A 125 CB A 1 CB 0 43 | A 368 CB A 1 CB 0 43 |  | EDC | | 1 | | 79 | | -0.11 |
| 29 | E139 | F1 |  | A 139 CB A 1 CB 0 43 | A 382 CB A 1 CB 0 43 |  | EDC | | 1 | | 9 | | -0.20 |
| 30 | E139 | K105 |  | A 139 CB A 105 CB 0 43 | A 382 CB A 105 CB 0 43 |  | EDC | | 1 | | 12 | | 0.38 |
| 31 | E139 | K240 |  | A 139 CB A 240 CB 0 43 | A 382 CB A 240 CB 0 43 |  | EDC | | 1 | | 13 | | -0.19 |
| 32 | E146 | F1 |  | A 146 CB A 1 CB 0 43 | A 389 CB A 1 CB 0 43 |  | EDC | | 1 | | 23 | | 0.54 |
| 33 | E146 | K240 |  | A 146 CB A 240 CB 0 43 | A 389 CB A 240 CB 0 43 |  | EDC | | 1 | | 31 | | -0.42 |
| 34 | D157 | F1 |  | A 157 CB A 1 CB 0 43 | A 400 CB A 1 CB 0 43 |  | EDC | | 1 | | 11 | | 0.17 |
| 35 | D157 | K240 |  | A 157 CB A 240 CB 0 43 | A 400 CB A 240 CB 0 43 |  | EDC | | 1 | | 20 | | -0.50 |
| 36 | D168 | F1 |  | A 168 CB A 1 CB 0 43 | A 411 CB A 1 CB 0 43 |  | EDC | | 1 | | 68 | | 0.14 |
| 37 | E198 | F1 |  | A 198 CB A 1 CB 0 43 | A 441 CB A 1 CB 0 43 |  | EDC | | 1 | | 26 | | 0.48 |
| 38 | E198 | K240 |  | A 198 CB A 240 CB 0 43 | A 441 CB A 240 CB 0 43 |  | EDC | | 1 | | 24 | | -0.53 |
| 39 | K213 | F1 |  | A 213 CB A 1 CB 0 43 | A 456 CB A 1 CB 0 43 |  | EDC | | 1 | | 31 | | 0.38 |
| 40 | K213 | K240 |  | A 213 CB A 240 CB 0 43 | A 456 CB A 240 CB 0 43 |  | EDC | | 1 | | 24 | | 0.31 |
| 41 | E234 | F1 |  | A 234 CB A 1 CB 0 43 | A 477 CB A 1 CB 0 43 |  | EDC | | 1 | | 47 | | 0.30 |
| 42 | E34 | F1 |  | A 34 CB A 1 CB 0 43 | A 277 CB A 1 CB 0 43 |  | EDC | | 1 | | 141 | | 0.30 |
| 43 | D73 | F1 |  | A 73 CB A 1 CB 0 43 | A 316 CB A 1 CB 0 43 |  | EDC | | 1 | | 126 | | 0.17 |
| 44 | E78 | F1 |  | A 78 CB A 1 CB 0 43 | A 321 CB A 1 CB 0 43 |  | EDC | | 1 | | 150 | | -0.07 |
| 45 | E78 | K240 |  | A 78 CB A 240 CB 0 43 | A 321 CB A 240 CB 0 43 |  | EDC | | 1 | | 25 | | 0.05 |
| 45 | D89 | F1 |  | A 89 CB A 1 CB 0 43 | A 332 CB A 1 CB 0 43 |  | EDC | | 1 | | 119 | | 0.13 |
| 47 | E92 | K240 |  | A 92 CB A 240 CB 0 43 | A 335 CB A 240 CB 0 43 |  | EDC | | 1 | | 10 | | 0.05 |

^a^ Chemical cross-links listed here were used for the DisVis and HADDOCK docking analysis.

^b^ Notation for the constraints file in the DisVis analysis for quantifying the information content of experimental cross-links in a potential interaction between APOA1 (fixed chain) and LCAT (scanning chain). The form is (APOA1 chain ID) (APOA1 residue #) (APOA1 atom identity – beta carbon) (LCAT chain ID) (LCAT residue #) (LCAT atom identity – β carbon) (minimal allowed distance between beta carbons in Å) (maximum allowed distance between beta carbons in Å). Since APOA1 exists as a reciprocating dimer on each disc, cross-links are indicated as possible for both APOA1 molecules A (residues 1-243) and B (residues 244-486). Cross-links that are underlined were evaluated in DisVis for potential binding site A while all others were evaluated for potential binding site B.

^c^ Variations of cross-linked peptides contributing to the identification. For example, if the same cross-link was found in multiple versions of peptides with skipped cleavages or was found with or without Met oxidation, etc.

^d^ Averaged ppm from all instances of identification across up to three independent experiments performed across about a 6 month period.

**DisVis false positive cross-linking constraint analysis:** DisVis is a software tool that allows the visualization and quantitation of the information content of a set of distance restraints describing the interaction between two protein structures, a fixed chain and a scanning chain ^2,3^. For our purposes, the fixed chain was human APOA1 that had been simulated as an rHDL particle with 160 molecules of POPC and 24 molecules of free cholesterol ^4^. In the PDB file, the two APOA1 molecules (243 a.a. each) were directly connected between the C-terminus of molecule A and the N-terminus of molecule B to give a continuous chain of amino acids from 1-486 because DisVis works with only two entities at a time. We began our initial analysis assuming that there is a one-to-one binding between LCAT and the dual APOA1 belt. However, because there are two reciprocating APOA1 belts, there are always two potential binding sites on either side of helix 5 for each cross-link detected. We called these putative sites A and B. The scanning chain was a model of human LCAT (4XWG) determined at 2.7 Å resolution^5^. A maximal span distance (between β carbons) was determined empirically for each cross-linker from studies of bovine serum albumin (see Supplementary Data). We used the average spacing for each cross-linker plus 1 standard deviation as constraints. These values were similar to those found in other studies of multiple cross-linker types ^6^. The cross-links in Table S1 appeared to cluster around two binding sites comprised of helix 4/6 on one side of the opposing helix 5 copies (Site A) and helix 6/4 on the other side (Site B). The cross-links in Table S1 that were closest to Site A were evaluated by DisVis separately from those closest to Site B.

**For site A**, the DisVis analysis showed that there were 73,998,042 accessible complexes with 0 constraints. This decreased to 0 possibilities with 20 of the restraints (19 restraints allowed for 1 possibility). The center of mass of the LCAT ligand with respect to the rHDL disc as a function of restraint is shown in **Fig. S1A**. The analysis generated a Z-score for each restraint. The higher the score, the more likely the restraint is a false positive. The results for putative binding site A are shown below. Cross-links in bold were judged compatible with a single binding site with a conservative Z-score cut-off of < -0.5:

**Table S2: Scoring of restraints for possible interactions between LCAT and APOA1 putative binding site A by DisVis**

| **#** | **Restraint** | **Avg violated fraction** | **S.D.** | **Z-score** |
| --- | --- | --- | --- | --- |
|  |  |  |  |  |
| **1** | **APOA1 116 – LCAT 240** | **0.41** | **0.34** | **-0.20** |
| *2* | *APOA1 123 – LCAT 240* | *0.46* | *0.30* | *0.02* |
| *3* | *APOA1 131 – LCAT 240* | *0.75* | *0.16* | *1.23* |
| **4** | **APOA1 106 – LCAT 240** | **0.31** | **0.28** | **-0.64** |
| **5** | **APOA1 107 – LCAT 240** | **0.26** | **0.29** | **-0.83** |
| **6** | **APOA1 118 – LCAT 116** | **0.36** | **0.29** | **-0.40** |
| **7** | **APOA1 118 – LCAT 240** | **0.24** | **0.29** | **-0.93** |
| *8* | *APOA1 78 – LCAT 240* | *0.71* | *0.26* | *1.04* |
| **9** | **APOA1 92 – LCAT 240** | **0.42** | **0.20** | **-0.17** |
| **10** | **APOA1 392 – LCAT 240** | **0.41** | **0.33** | **-0.22** |
| **11** | **APOA1 394 – LCAT 240** | **0.36** | **0.34** | **-0.43** |
| **12** | **APOA1 396 – LCAT 240** | **0.43** | **0.33** | **-0.13** |
| *13* | *APOA1 414 – LCAT 240* | *0.74* | *0.26* | *1.17* |
| *14* | *APOA1 416 – LCAT 240* | *0.76* | *0.27* | *1.28* |
| *15* | *APOA1 376 – LCAT 240* | *0.47* | *0.27* | *0.05* |
| **16** | **APOA1 383 – LCAT 240** | **0.29** | **0.28** | **-0.70** |
| **17** | **APOA1 382 – LCAT 105** | **0.39** | **0.24** | **-0.31** |
| **18** | **APOA1 382 – LCAT 240** | **0.17** | **0.23** | **-1.22** |
| **19** | **APOA1 389 – LCAT 240** | **0.12** | **0.21** | **-1.40** |
| **20** | **APOA1 400 – LCAT 240** | **0.15** | **0.22** | **-1.28** |
| *21* | *APOA1 441 – LCAT 240* | *0.93* | *0.12* | *1.96* |
| *22* | *APOA1 456 – LCAT 240* | *0.96* | *0.08* | *2.10* |

Note: Cross-links involving the amino terminal amino group of LCAT residue 1 (shown in Table S1) were not included in the DisVis analysis because the N-terminal 19 residues are not visible in the crystal structure.

We used an arbitrary cut-off of 0 for the Z-score for cross-links to be used during docking analyses. Thus, 14 of the 22 cross-links (bold in Table S2) were judged to be consistent with LCAT binding to Site A.


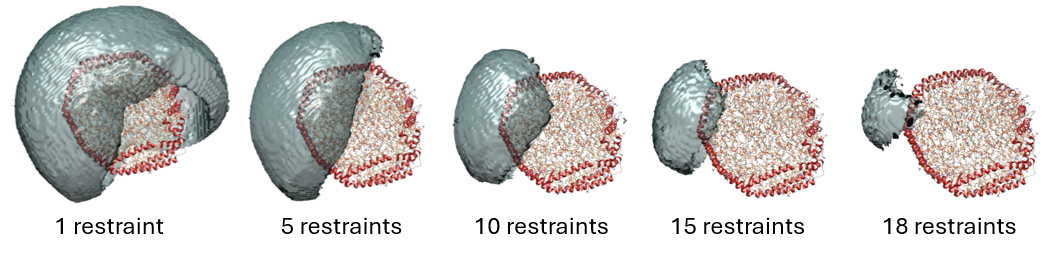


**Figure S1**. The accessible interaction space consistent with at least N restraints. The gray area shows possible locations of the center of mass of LCAT with respect to the rHDL disc for binding site A.

**For Site B**, there were 74,236,527 accessible complexes with 0 constraints. This decreased to 0 possibilities with 19 of the restraints (18 restraints allowed for 4 possibilities). The results for site B are shown below:

**Table S3: Scoring of restraints for possible interactions between LCAT and APOA1 putative binding site B by DisVis**

| **#** | **Restraint** | **Avg violated fraction** | **S.D.** | **Z-score** |
| --- | --- | --- | --- | --- |
|  |  |  |  |  |
| **1** | **APOA1 149– LCAT 240** | **0.44** | **0.34** | **-0.17** |
| **2** | **APOA1 151 – LCAT 240** | **0.39** | **0.36** | **-0.39** |
| **3** | **APOA1 153 – LCAT 240** | **0.46** | **0.32** | **-0.07** |
| *4* | *APOA1 171 – LCAT 240* | *0.78* | *0.20* | *1.29* |
| *5* | *APOA1 173 – LCAT 240* | *0.83* | *0.09* | *1.50* |
| *6* | *APOA1 133 – LCAT 116* | *0.49* | *0.21* | *0.06* |
| **7** | **APOA1 140 – LCAT 240** | **0.32** | **0.28** | **-0.69** |
| **8** | **APOA1 139 – LCAT 105** | **0.43** | **0.23** | **-0.21** |
| **9** | **APOA1 139 – LCAT 240** | **0.19** | **0.23** | **-1.24** |
| **10** | **APOA1 146 – LCAT 240** | **0.14** | **0.22** | **-1.45** |
| **11** | **APOA1 157 – LCAT 240** | **0.16** | **0.22** | **-1.36** |
| *12* | *APOA1 198 – LCAT 240* | *0.93* | *0.11* | *1.91* |
| *13* | *APOA1 213 – LCAT 240* | *0.91* | *0.10* | *1.85* |
| **14** | **APOA1 359 – LCAT 240** | **0.43** | **0.33** | **-0.19** |
| *15* | *APOA1 366 – LCAT 240* | *0.51* | *0.29* | *0.15* |
| *16* | *APOA1 374 – LCAT 240* | *0.75* | *0.16* | *1.14* |
| **17** | **APOA1 349 – LCAT 105** | **0.31** | **0.29** | **-0.70** |
| **18** | **APOA1 350 – LCAT 240** | **0.27** | **0.30** | **-0.89** |
| **19** | **APOA1 361 – LCAT 116** | **0.40** | **0.30** | **-0.35** |
| **20** | **APOA1 361 – LCAT 240** | **0.26** | **0.30** | **-0.95** |
| *21* | *APOA1 321 – LCAT 240* | *0.72* | *0.20* | *1.04* |
| **22** | **APOA1 335 – LCAT 240** | **0.42** | **0.17** | **-0.26** |

Thus, 14 of the 22 cross-links (bold in Table S3) were judged to be consistent with LCAT binding to Site A.


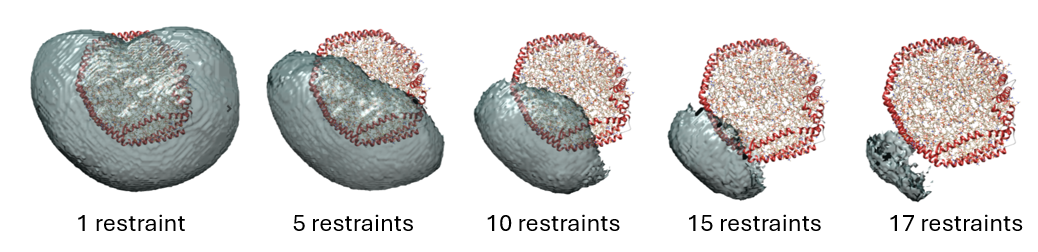


**Figure S2**. The accessible interaction space consistent with at least N restraints. The gray area shows possible locations of the center of mass of LCAT with respect to the rHDL disc for binding site B. Note that the rHDL disc is oriented the same as in Fig. S1.

Note: We performed the same DisViz analysis using the ‘open’ LCAT structure 6MVD^7^, which was derived in the presence of an activating small molecule and thought to represent an active conformation of LCAT. The Z-scores were similar to those for 4XWG.

**HADDOCK docking analysis:** HADDOCK (High Ambiguity Driven protein-protein DOCKing) is an algorithm that uses a variety of experimentally derived restraints to drive docking simulations ^8,9^. It can incorporate distance restraints from chemical cross-linking experiments as well as electron density envelopes from cryo-EM studies. We used the HADDOCK 2.4 portal to analyze the interactions between the APOA1 rHDL particle simulation structure (described above, molecule A) and two copies of the crystal structure of LCAT (molecules B and C). We ran parallel analyses using two crystal structures of LCAT (4XWG and 6MVD). The rHDL PDB file was modified to list all lipids as HETATMs and so they did not conflict with protein residue and chain names. Additionally, all hydrogens were removed from the structure to improve computation speeds. We noticed no difference in results when hydrogens were present. Prior to the HADDOCK analysis, the rHDL particle and each LCAT molecule were separately fit into both the flipped and unflipped cryo-EM envelopes (as mrc files) using PowerFit ^2^. The resulting centroid coordinates were used as ambiguous restraints for the docking along with the cryo-EM envelopes mrc file. We also ran two combinations of the rHDL disc called “up” and “down” distinguished the position of the hairpin N-terminal domain on one side of the simulated disc. This resulted in 8 possible docking combinations that needed to be analyzed. The rHDL particle was restricted to the measured centroid coordinates of the appropriate map during the it0 phase of the docking run while the LCAT molecules were left unrestricted. Molecule types were set to “Protein or Protein-ligand”. Course-grain was disabled.


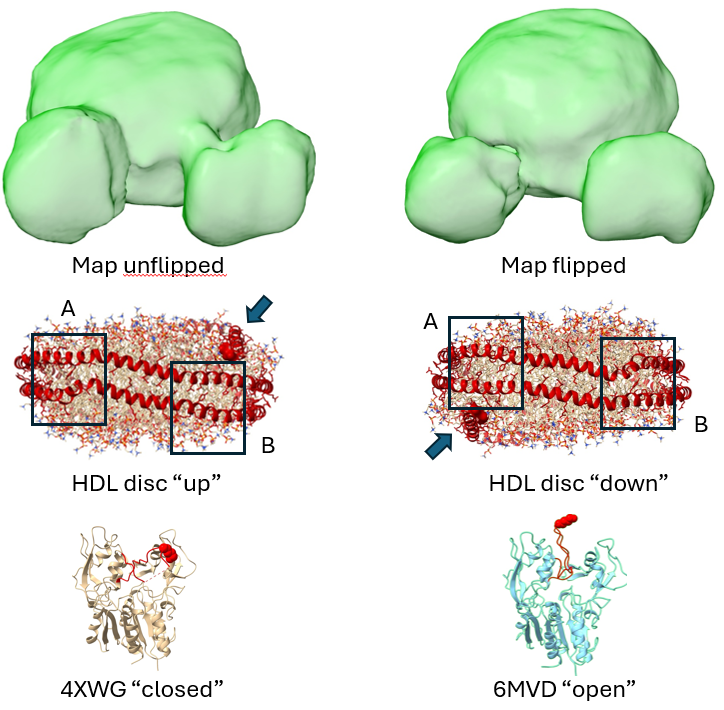


**Figure S3**. The docking analysis was performed with both flipped and unflipped maps, two different crystal structures of LCAT (4XWG which has a ‘closed’ lid domain shown in red with residue 240 shown in spacefill, and 6MVD which has an open lid shown in the same color scheme. Because the APOA1 double belts were not directly modeled in the center of the lipid bilayer, the rHDL particle was tested in both an ‘up’ and ‘down’ orientation as distinguished by the back-looped N-terminus present on one side of the particle (see arrows).

The DisVis analysis on the experimental cross-links described above provided for at least 2 potential LCAT binding sites (A and B). To analyze binding to these two sites with the rHDL up and rHDL down target, two separate restraint files were required.

**Restraints, rHDL “up”:**

assign (resid 116 and segid A and name CB)(resid 240 and segid B and name CB) 27 27 0

assign (resid 106 and segid A and name CB)(resid 240 and segid B and name CB) 33 33 0

assign (resid 107 and segid A and name CB)(resid 240 and segid B and name CB) 33 33 0

assign (resid 131 and segid A and name CB)(resid 240 and segid B and name CB) 27 27 0

assign (resid 118 and segid A and name CB)(resid 116 and segid B and name CB) 33 33 0

assign (resid 118 and segid A and name CB)(resid 240 and segid B and name CB) 33 33 0

assign (resid 92 and segid A and name CB)(resid 240 and segid B and name CB) 43 43 0

assign (resid 392 and segid A and name CB)(resid 240 and segid B and name CB) 27 27 0

assign (resid 394 and segid A and name CB)(resid 240 and segid B and name CB) 27 27 0

assign (resid 396 and segid A and name CB)(resid 240 and segid B and name CB) 27 27 0

assign (resid 383 and segid A and name CB)(resid 240 and segid B and name CB) 33 33 0

assign (resid 382 and segid A and name CB)(resid 240 and segid B and name CB) 43 43 0

assign (resid 382 and segid A and name CB)(resid 105 and segid B and name CB) 43 43 0

assign (resid 389 and segid A and name CB)(resid 240 and segid B and name CB) 43 43 0

assign (resid 400 and segid A and name CB)(resid 240 and segid B and name CB) 43 43 0

assign (resid 149 and segid A and name CB)(resid 240 and segid C and name CB) 27 27 0

assign (resid 151 and segid A and name CB)(resid 240 and segid C and name CB) 27 27 0

assign (resid 153 and segid A and name CB)(resid 240 and segid C and name CB) 27 27 0

assign (resid 140 and segid A and name CB)(resid 240 and segid C and name CB) 43 43 0

assign (resid 139 and segid A and name CB)(resid 105 and segid C and name CB) 43 43 0

assign (resid 139 and segid A and name CB)(resid 240 and segid C and name CB) 43 43 0

assign (resid 146 and segid A and name CB)(resid 240 and segid C and name CB) 43 43 0

assign (resid 157 and segid A and name CB)(resid 240 and segid C and name CB) 43 43 0

assign (resid 359 and segid A and name CB)(resid 240 and segid C and name CB) 27 27 0

assign (resid 349 and segid A and name CB)(resid 240 and segid C and name CB) 33 33 0

assign (resid 350 and segid A and name CB)(resid 240 and segid C and name CB) 33 33 0

assign (resid 361 and segid A and name CB)(resid 116 and segid C and name CB) 33 33 0

assign (resid 361 and segid A and name CB)(resid 240 and segid C and name CB) 33 33 0

assign (resid 335 and segid A and name CB)(resid 240 and segid C and name CB) 33 33 0

**Restraints, rHDL “down”:**

assign (resid 116 and segid A and name CB)(resid 240 and segid C and name CB) 27 27 0

assign (resid 123 and segid A and name CB)(resid 240 and segid C and name CB) 27 27 0

assign (resid 131 and segid A and name CB)(resid 240 and segid C and name CB) 27 27 0

assign (resid 118 and segid A and name CB)(resid 116 and segid C and name CB) 33 33 0

assign (resid 118 and segid A and name CB)(resid 240 and segid C and name CB) 33 33 0

assign (resid 392 and segid A and name CB)(resid 240 and segid C and name CB) 27 27 0

assign (resid 394 and segid A and name CB)(resid 240 and segid C and name CB) 27 27 0

assign (resid 396 and segid A and name CB)(resid 240 and segid C and name CB) 27 27 0

assign (resid 383 and segid A and name CB)(resid 240 and segid C and name CB) 33 33 0

assign (resid 382 and segid A and name CB)(resid 240 and segid C and name CB) 43 43 0

assign (resid 382 and segid A and name CB)(resid 105 and segid C and name CB) 43 43 0

assign (resid 389 and segid A and name CB)(resid 240 and segid C and name CB) 43 43 0

assign (resid 400 and segid A and name CB)(resid 240 and segid C and name CB) 43 43 0

assign (resid 149 and segid A and name CB)(resid 240 and segid B and name CB) 27 27 0

assign (resid 151 and segid A and name CB)(resid 240 and segid B and name CB) 27 27 0

assign (resid 153 and segid A and name CB)(resid 240 and segid B and name CB) 27 27 0

assign (resid 140 and segid A and name CB)(resid 240 and segid B and name CB) 43 43 0

assign (resid 139 and segid A and name CB)(resid 240 and segid B and name CB) 43 43 0

assign (resid 146 and segid A and name CB)(resid 240 and segid B and name CB) 43 43 0

assign (resid 157 and segid A and name CB)(resid 240 and segid B and name CB) 43 43 0

assign (resid 359 and segid A and name CB)(resid 240 and segid B and name CB) 27 27 0

assign (resid 349 and segid A and name CB)(resid 240 and segid B and name CB) 33 33 0

assign (resid 350 and segid A and name CB)(resid 240 and segid B and name CB) 33 33 0

assign (resid 361 and segid A and name CB)(resid 116 and segid B and name CB) 33 33 0

assign (resid 361 and segid A and name CB)(resid 240 and segid B and name CB) 33 33 0

assign (resid 335 and segid A and name CB)(resid 240 and segid B and name CB) 33 33 0

The highest scoring docking model from each of the combinations is shown below:


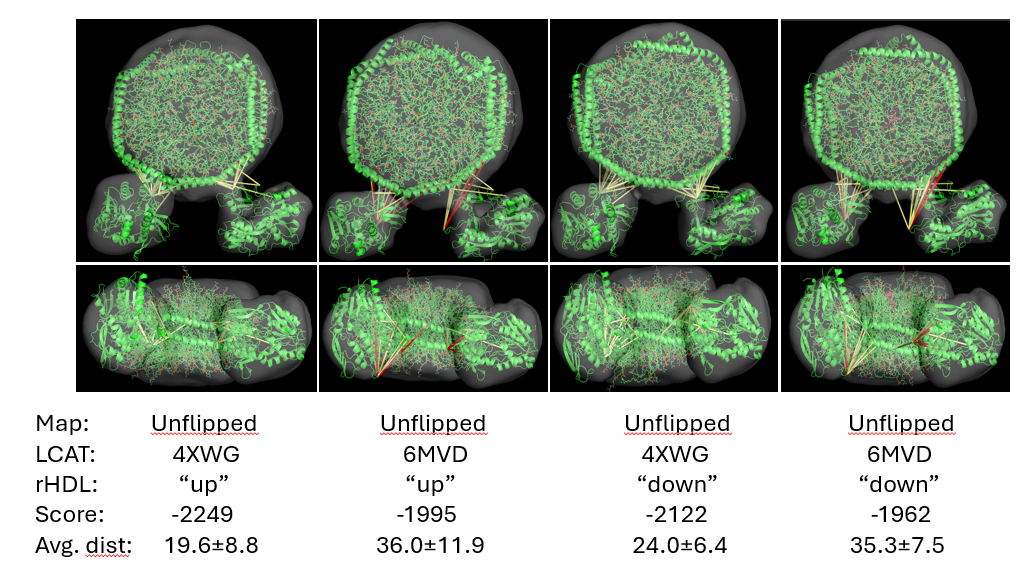


**Figure S4**. Docking analysis results for the unflipped map. **Top:** each of the combinations is shown “top down” with helix 5 oriented toward the bottom of the figure. Bottom: side view looking directly at helix 5. Cross-links are shown in yellow. Cross-links shown in red exceeded the allowed spacer arm length specified in the restraint files listed above.

| **HADDOCK Parameter** | **Combination 1** | **Combination 2** | **Combination 3** | **Combination 4** |
| --- | --- | --- | --- | --- |
| EM Map used | unflipped | unflipped | unflipped | unflipped |
| LCAT structure used | 4XWG | 6MVD | 4XWG | 6MVD |
| rHDL orientation | “up” | “up” | “down” | “down” |
| HADDOCK Score | -2249 ± 69 | -1995 ± 20 | -2122 ± 27 | -1962 ± 270.7 |
| Cluster size | 8 | 140 | 188 | 200 |
| Van der Waals energy | -18.1 ± 3.6 | -58.8 ± 10.7 | -49.6 ± 13.5 | -75.3 ± 11.7 |
| Electrostatic energy | -115.7 ± 51.7 | -172.0 ± 33.7 | -109.3 ± 54.5 | -169.9 ± 29.4 |
| Desolvation energy | 1.5 ± 6.1 | -1.3 ± 4.2 | -6.2 ± 2.9 | -6.0 ± 4.9 |
| Restraint violation energy | 38.6 ± 22.8 | 1454.3 ± 25.6 | 92.0 ± 12.8 | 1051.4 ± 13.9 |
| Buried surface area | 774.2 ± 105.7 | 2176.2 ± 166.0 | 1674.6 ± 133.4 | 2866.6 ± 192.2 |
| Avg. XL distance (Cβ-Cβ in Å) | 19.6 ± 8.8 | 36.0 ± 11.9 | 24.0 ± 6.4 | 35.3 ± 7.5 |


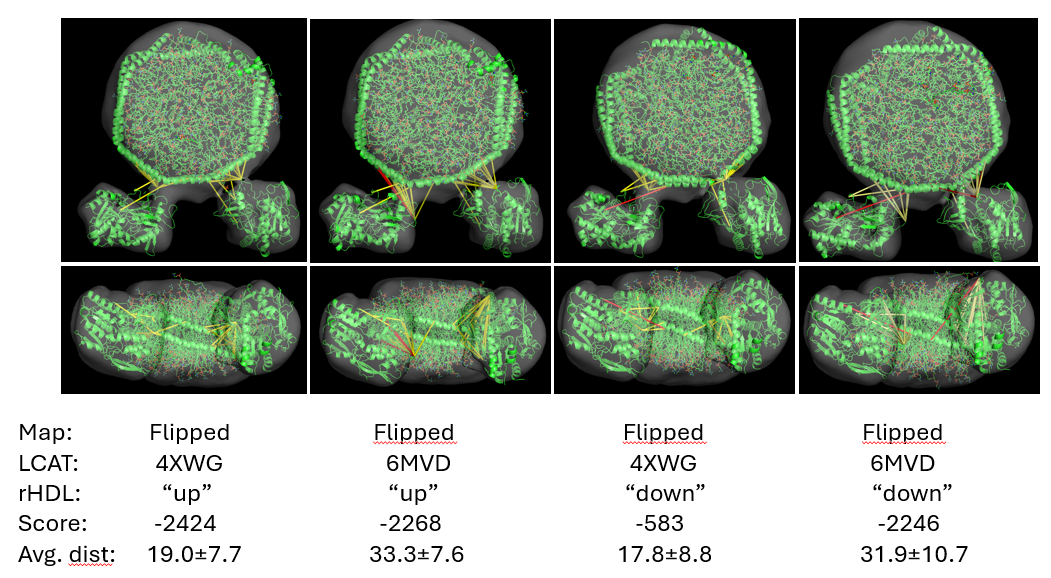


**Figure S5**. Docking analysis results for the flipped map. **Top:** each of the combinations is shown “top down” with helix 5 oriented toward the bottom of the figure. **Bottom:** side view looking directly at helix 5. Cross-links are shown in yellow. Cross-links shown in red exceeded the allowed spacer arm length specified in the restraint files listed above.

| **HADDOCK Parameter** | **Combination 5** | **Combination**  **6** | **Combination 7** | **Combination 8** |
| --- | --- | --- | --- | --- |
| EM Map used | flipped | flipped | flipped | flipped |
| LCAT structure used | 4XWG | 6MVD | 4XWG | 6MVD |
| rHDL orientation | “up” | “up” | “down” | “down” |
| HADDOCK Score | -2424 ± 42 | -2268 ± 19 | -583 ± 26 | -2246 ± 23 |
| Cluster size | 177 | 157 | 200 | 187 |
| Van der Waals energy | -24.8 ± 4.2 | -27.2 ± 3.5 | 1757.9 ± 6.8 | -32.5 ± 5.2 |
| Electrostatic energy | -88.9 ± 36.8 | -55.8 ± 8.2 | -257.9 ± 46.9 | 0.3 ± 8.4 |
| Desolvation energy | -1.4 ± 5.0 | -2.2 ± 2.8 | 12.1 ± 5.1 | -13.8 ± 1.7 |
| Restraint violation energy | 0.0 ± 0.0 | 868.3 ± 62.4 | 1.6 ± 1.1 | 878.7 ± 20.6 |
| Buried surface area | 990 ± 133 | 1088.0 ± 29.8 | 1306.0 ± 86.3 | 1177.4 ± 164.1 |
| Avg. XL distance (Cβ-Cβ in Å) | 19.0 ± 7.7 | 33.3 ± 7.6 | 17.8 ± 8.8 | 31.9 ± 10.7 |

**References:**

1 Tayri-Wilk, T. *et al.* Mass spectrometry reveals the chemistry of formaldehyde cross-linking in structured proteins. *Nat Commun* **11**, 3128, doi:10.1038/s41467-020-16935-w (2020).

2 van Zundert, G. C. *et al.* The DisVis and PowerFit Web Servers: Explorative and Integrative Modeling of Biomolecular Complexes. *J Mol Biol* **429**, 399-407, doi:10.1016/j.jmb.2016.11.032 (2017).

3 van Zundert, G. C. & Bonvin, A. M. DisVis: quantifying and visualizing accessible interaction space of distance-restrained biomolecular complexes. *Bioinformatics* **31**, 3222-3224, doi:10.1093/bioinformatics/btv333 (2015).

4 Pourmousa, M. *et al.* Tertiary structure of apolipoprotein A-I in nascent high-density lipoproteins. *Proc Natl Acad Sci U S A* **115**, 5163-5168, doi:10.1073/pnas.1721181115 (2018).

5 Piper, D. E. *et al.* The high-resolution crystal structure of human LCAT. *J Lipid Res* **56**, 1711-1719, doi:10.1194/jlr.M059873 (2015).

6 Leitner, A. *et al.* Chemical cross-linking/mass spectrometry targeting acidic residues in proteins and protein complexes. *Proc Natl Acad Sci U S A* **111**, 9455-9460, doi:10.1073/pnas.1320298111 (2014).

7 Manthei, K. A. *et al.* A retractable lid in lecithin:cholesterol acyltransferase provides a structural mechanism for activation by apolipoprotein A-I. *J Biol Chem* **292**, 20313-20327, doi:10.1074/jbc.M117.802736 (2017).

8 Honorato, R. V. *et al.* Structural Biology in the Clouds: The WeNMR-EOSC Ecosystem. *Front Mol Biosci* **8**, 729513, doi:10.3389/fmolb.2021.729513 (2021).

9 Dominguez, C., Boelens, R. & Bonvin, A. M. HADDOCK: a protein-protein docking approach based on biochemical or biophysical information. *J Am Chem Soc* **125**, 1731-1737, doi:10.1021/ja026939x (2003).
