# Supplementary material for: Lecithin:cholesterol acyltransferase binds a discontinuous binding site on adjacent apolipoprotein A-I belts in HDL
Source: J Lipid Res. 2025 Mar 25;66(5):100786. doi: 10.1016/j.jlr.2025.100786 (PMC12049944; doi:10.1016/j.jlr.2025.100786)
Supplement: LCAT Manuscript Data Supplement New [file mmc2.docx]

**Lecithin:Cholesterol Acyltransferase Interacts With a Discontinuous Binding Site on Adjacent Apolipoprotein A-I Belts in High Density Lipoproteins**

**Online Data Supplement**

Bethany Coleman^1^, Shimpi Bedi^2^, John H. Hill^3^, Jamie Morris^2^, Kelly A. Manthei^4^, Rachel C. Hart^5^, Yi He^6^, Amy S. Shah^7^, W. Gray Jerome^5^, Tomas Vaisar^6^, Karin E. Bornfeldt^6^, Hyun Song^8^, Jere P. Segrest^8^, Jay W. Heinecke^6^, Stephen G. Aller^3^, John J. G. Tesmer^9^ and W. Sean Davidson^2*^

^1^Department of Molecular and Cellular Biosciences, University of Cincinnati, Cincinnati OH 45237

^2^Department of Pathology and Laboratory Medicine, University of Cincinnati, Cincinnati OH 45237

^3^ Department of Pharmacology and Toxicology, University of Alabama at Birmingham, Birmingham, Alabama 35294

^4^ Life Sciences Institute, University of Michigan, Ann Arbor, Michigan 48109

^5^ Department of Pathology, Microbiology and Immunology, Vanderbilt University School of Medicine, Nashville, Tennessee 37232

^6^ Department of Medicine, University of Washington School of Medicine, Seattle, Washington 98109

^7^ Department of Pediatrics, Cincinnati Children’s Hospital Medical Center and the University of Cincinnati, Cincinnati, OH 45229

^8^ Department of Medicine, Vanderbilt University Medical Center, Nashville, Tennessee 37232

^9^ Departments of Biological Sciences and Medicinal Chemistry and Molecular Pharmacology, Purdue University, West Lafayette, IN, 47907

| **APOA1 Mutation** | **Location in Human APOA1** | **Reason for Targeting** | **Produced Acceptable rHDL vs. WT?** |
| --- | --- | --- | --- |
| K96A | Helix 3 | Control mutation in Helix 3 - outside hypothesized 4/6 helical binding site | Yes |
| P99A | Helix 4 | Initiating proline of helix 4 - if its loss disrupts helix, may affect LCAT activity | **No** |
| Y100A | Helix 4 | Highly species conserved residue in helix 4 | Yes |
| Q105A | Helix 4 | Highly species conserved residue in helix 4 | Yes |
| K106A | Helix 4 | Polar residue exposed in helix 4 in models, not highly conserved. Alanine will take away the charge | Yes |
| K107A | Helix 4 | Highly conserved polar residue in helix 4. Alanine will take away the charge | Yes |
| W108A | Helix 4 | Highly conserved hydrophobic and bulky residue in helix 4. Alanine will reduce hydrophobicity and bulk | Yes |
| W108K | Helix 4 | Highly conserved hydrophobic and bulky residue in helix 4. Lysine will put a positive charge there. May disrupt helix | **No** |
| E110A | Helix 4 | Highly conserved acidic residue in helix 4. Alanine substitution will eliminate the charge | Yes |
| E111A | Helix 4 | Highly conserved acidic residue in helix 4. Alanine substitution will eliminate the charge | Yes |
| E111K | Helix 4 | Highly conserved acidic residue in helix 4. Lysine substitution will reverse the charge | **No** |
| Y115A | Helix 4 | Highly conserved in helix 4. Alanine substitution will reduce volume | Yes |
| K118A | Helix 4 | Exposed and highly conserved basic residue in helix 4. Alanine will eliminate charge | Yes |
| E147A | Helix 6 | Reasonably conserved acidic residue in helix 6. Alanine will eliminate charge | Yes |
| R149A | Helix 6 | Highly conserved basic residue in helix 6. Alanine will eliminate charge | **No** |
| R151A | Helix 6 | Highly conserved basic residue in helix 6. Alanine will eliminate charge | Yes |
| R153A | Helix 6 | Highly conserved basic residue in helix 6. Alanine will eliminate charge | Yes |
| H155A | Helix 6 | Reasonably conserved basic residue in helix 6. Alanine will eliminate charge | **No** |
| V156E | Helix 6 | Highly conserved small hydrophobic residue in helix 6. Glutamic acid will put a negative charge there with similar bulk | Yes |
| A158K | Helix 6 | Poorly conserved low hydrophobicity residue in helix 6. Lysine will put more bulk and a charge there. Do not expect much effect on LCAT activity. | Yes |
| L159K | Helix 6 | Highly conserved small hydrophobic residue in helix 6. Arginine mutation here exhibits elevated triglycerides, low APOA1 and HDL-C (Fin) | Yes |
| E191A | Helix 8 | Control mutation in Helix 8 outside the hypothesized 4/6 helical binding site. Do not expect much effect on LCAT activity. | Yes |

**Supplementary Table 1:** **Mutations in human APOA1 aimed at testing the hypothesis that residues in both helices 4 and 6 can affect LCAT activity.** The amino acid substitution made, the helical location, our pre-experimental rationale for targeting this site, and whether or not the mutant generated rHDL particles of similar size and composition to those made with WT APOA1 are shown.

**Supplement Figure S1**


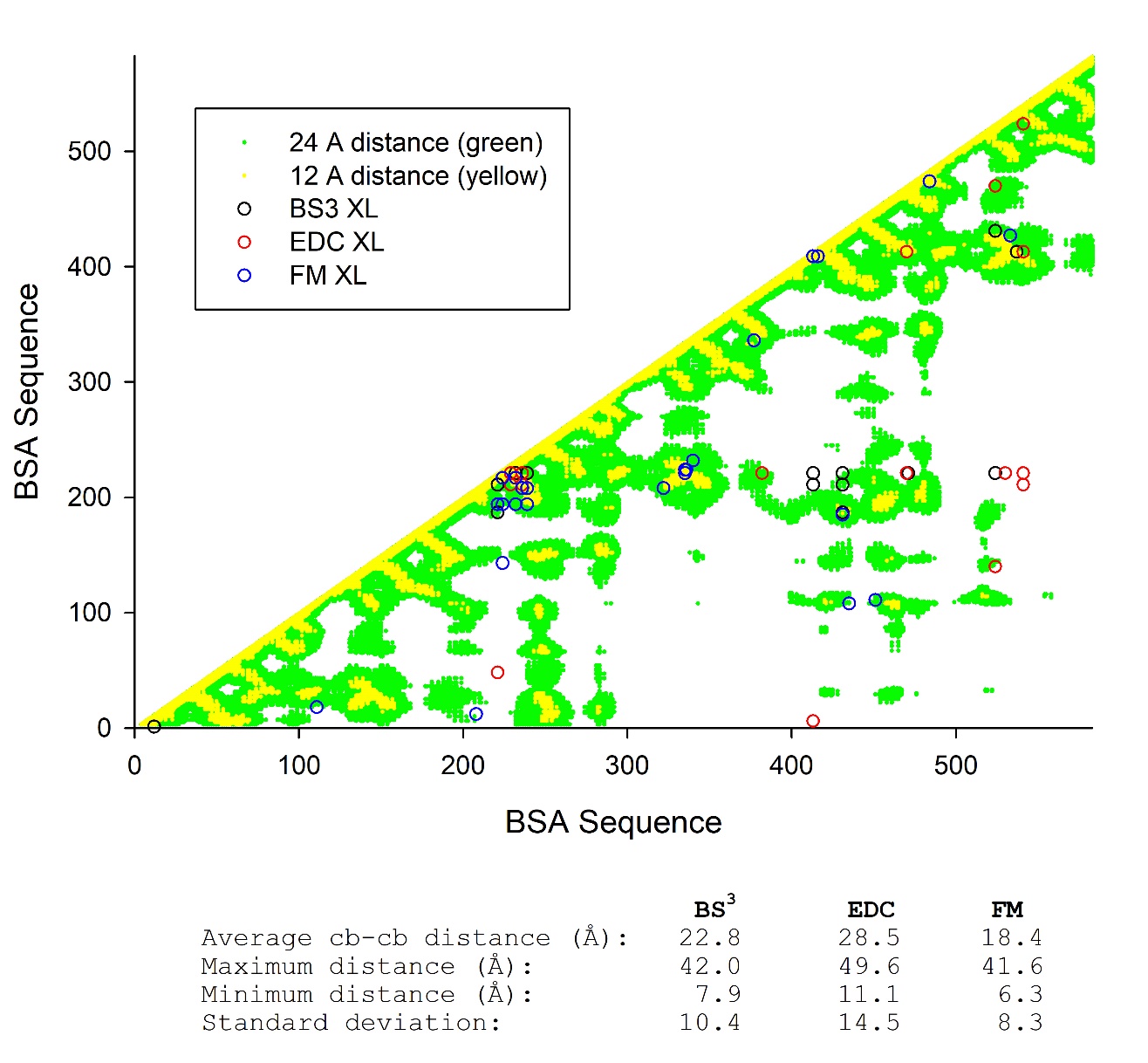


**Figure S1**: **Cross-linker evaluation using bovine serum albumin, a well-known soluble protein structure**. Fatty acid free bovine serum albumin in phosphate-buffered saline was incubated with BS^3^, EDC, and FM under conditions described in *Methods*. After reaction quenching, the proteins were exhaustively digested with trypsin and the resulting peptides were analyzed by nano-spray MS analysis. MS and MS/MS data from triplicate experiments were analyzed by pLink at a 1% FDR with a 5 ppm cut-off. Our criteria for cross-link inclusion are laid out in *Methods*. Using the crystal structure of BSA (3VO3), we generated a contact plot ^1^ showing the proximity of residues (alpha-carbons) within 12 Å (yellow) and 24Å (green). All areas in white are > 24 Å. Identified cross-links using each reagent (BS^3^, black circles; EDC, red circles; FM blue circles) are shown superimposed on the contact plot. In general, most cross-links fell on the yellow or green areas of the contact plot indicating spacing distances of > 24 Å. However, several appeared to exceed this distance, despite being reliably identified. Actual distances in 3VO3 were measured in PyMol and the parameters are shown in the table. These experimentally derived spacer arm lengths were used as guidance in the docking analysis of LCAT to APOA1 in rHDL. The contact map data was generated using GNUPLOT (www.gnuplot.info) and then plotted using SigmaPlot 11.2 (Grafiti, Palo Alto, CA).

**Supplement Figure S2**


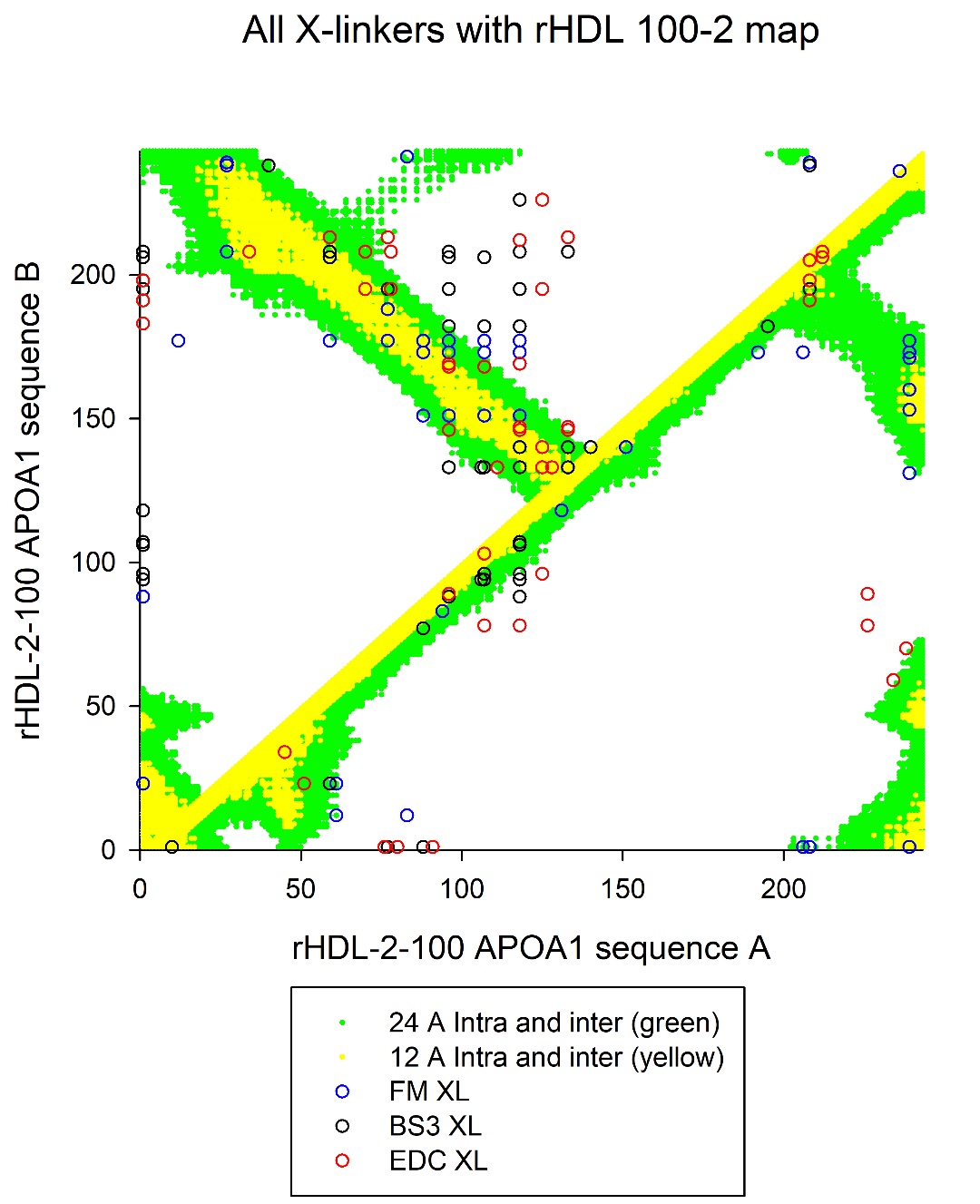


**Figure S2**: **APOA1 to APOA1 cross-links in rHDL incubated with LCAT**. rHDL particles were incubated with human LCAT and subjected to chemical cross-linking with BS^3^, EDC and FM under conditions described in *Methods*. After reaction quenching, the proteins were exhaustively digested with trypsin and the resulting peptides were analyzed by nano-spray MS analysis. MS and MS/MS data from triplicate experiments were analyzed by pLink at a 1% FDR with a 5 ppm cut-off. Our criteria for cross-link inclusion are laid out in *Methods*. Using the simulated structure of a rHDL-2-100 particle, ^2^ we generated a contact plot showing the proximity of residues (α-carbons) within 12 Å (yellow) and 24 Å (green). All areas in white are > 24 Å. Because there are two molecules of APOA1 in the particles, intra-molecular contacts are shown on the lower right half of the plot while inter-molecular contacts are shown on the upper left half. Identified cross-links using each reagent (BS^3^, black circles; EDC, red circles; FM blue circles) are shown superimposed on the contact plot. In the case of the FM cross-links, intra- versus inter-molecular crosslinks were verified by isotope-assisted cross-linking ^3^. For BS^3^ and EDC, mono-isotopic experiments were performed, and the molecular span was assigned as best fit to the contact plot. In general, most cross-links fell on the yellow or green areas of the contact plot indicating spacing distances of < 24 Å.

**Supplementary Table 2: Chemical cross-links between APOA1-containing rHDL and human LCAT.**

|  | **Residue^a^** | |  |  |  |  | |  | |
| --- | --- | --- | --- | --- | --- | --- | --- | --- | --- |
| **#** | **APOA1** | **LCAT** |  |  | **Linker** | **Forms^a^** | **Hits^b^** | | **ppm^c^** |
| 1 | R116 | K240 |  |  | FM | 2 | 71 | | -0.35 |
| 2 | R123 | K240 |  |  | FM | 1 | 35 | | -0.26 |
| 3 | R131 | K240 |  |  | FM | 1 | 76 | | -0.67 |
| 4 | R149 | K240 |  |  | FM | 2 | 17 | | -0.61 |
| 5 | R151 | K240 |  |  | FM | 1 | 90 | | -0.61 |
| 6 | R153 | K240 |  |  | FM | 1 | 54 | | -0.76 |
| 7 | R171 | K240 |  |  | FM | 1 | 89 | | -0.04 |
| 8 | R173 | K240 |  |  | FM | 1 | 26 | | -0.82 |
| 9 | K106 | F1 |  |  | BS^3^ | 1 | 32 | | 0.51 |
| 10 | K106 | K240 |  |  | BS^3^ | 1 | 12 | | -0.61 |
| 11 | K107 | F1 |  |  | BS^3^ | 2 | 131 | | -0.05 |
| 12 | K107 | K240 |  |  | BS^3^ | 1 | 21 | | 0.28 |
| 13 | K118 | F1 |  |  | BS^3^ | 1 | 179 | | -0.07 |
| 14 | K118 | K116 |  |  | BS^3^ | 1 | 36 | | -0.73 |
| 15 | K118 | K240 |  |  | BS^3^ | 1 | 33 | | -0.29 |
| 16 | K133 | F1 |  |  | BS^3^ | 1 | 221 | | -0.08 |
| 17 | K133 | K240 |  |  | BS^3^ | 1 | 23 | | -0.98 |
| 18 | K140 | F1 |  |  | BS^3^ | 2 | 348 | | -0.70 |
| 19 | K140 | K240 |  |  | BS^3^ | 1 | 17 | | -0.89 |
| 20 | K182 | F1 |  |  | BS^3^ | 1 | 121 | | 0.12 |
| 21 | K195 | F1 |  |  | BS^3^ | 1 | 61 | | 0.01 |
| 22 | K206 | F1 |  |  | BS^3^ | 1 | 65 | | 0.28 |
| 23 | K208 | F1 |  |  | BS^3^ | 1 | 63 | | 0.17 |
| 24 | K88 | F1 |  |  | BS^3^ | 2 | 57 | | 0.35 |
| 25 | K94 | F1 |  |  | BS^3^ | 1 | 597 | | 0.32 |
| 26 | K96 | F1 |  |  | BS^3^ | 1 | 313 | | 0.22 |
| 27 | E111 | F1 |  |  | EDC | 1 | 48 | | -0.01 |
| 28 | E125 | F1 |  |  | EDC | 1 | 79 | | -0.11 |
| 29 | E139 | F1 |  |  | EDC | 1 | 9 | | -0.20 |
| 30 | E139 | K105 |  |  | EDC | 1 | 12 | | 0.38 |
| 31 | E139 | K240 |  |  | EDC | 1 | 13 | | -0.19 |
| 32 | E146 | F1 |  |  | EDC | 1 | 23 | | 0.54 |
| 33 | E146 | K240 |  |  | EDC | 1 | 31 | | -0.42 |
| 34 | D157 | F1 |  |  | EDC | 1 | 11 | | 0.17 |
| 35 | D157 | K240 |  |  | EDC | 1 | 20 | | -0.50 |
| 36 | D168 | F1 |  |  | EDC | 1 | 68 | | 0.14 |
| 37 | E198 | F1 |  |  | EDC | 1 | 26 | | 0.48 |
| 38 | E198 | K240 |  |  | EDC | 1 | 24 | | -0.53 |
| 39 | K213 | F1 |  |  | EDC | 1 | 31 | | 0.38 |
| 40 | K213 | K240 |  |  | EDC | 1 | 24 | | 0.31 |
| 41 | E234 | F1 |  |  | EDC | 1 | 47 | | 0.30 |
| 42 | E34 | F1 |  |  | EDC | 1 | 141 | | 0.30 |
| 43 | D73 | F1 |  |  | EDC | 1 | 126 | | 0.17 |
| 44 | E78 | F1 |  |  | EDC | 1 | 150 | | -0.07 |
| 45 | E78 | K240 |  |  | EDC | 1 | 25 | | 0.05 |
| 45 | D89 | F1 |  |  | EDC | 1 | 119 | | 0.13 |
| 47 | E92 | K240 |  |  | EDC | 1 | 10 | | 0.05 |

^a^ Variations of cross-linked peptides contributing to the identification. For example, the same cross-link was found in multiple versions of peptides with skipped cleavages or was found with or without Met oxidation, etc.

^b^ Number of times the peptide containing the cross-link was identified by MS/MS, a rough but imperfect indication of abundance.

^c^ Averaged parts per million from all instances of identification across up to three independent experiments performed across about a 6-month period.

**Supplement Figure S3**


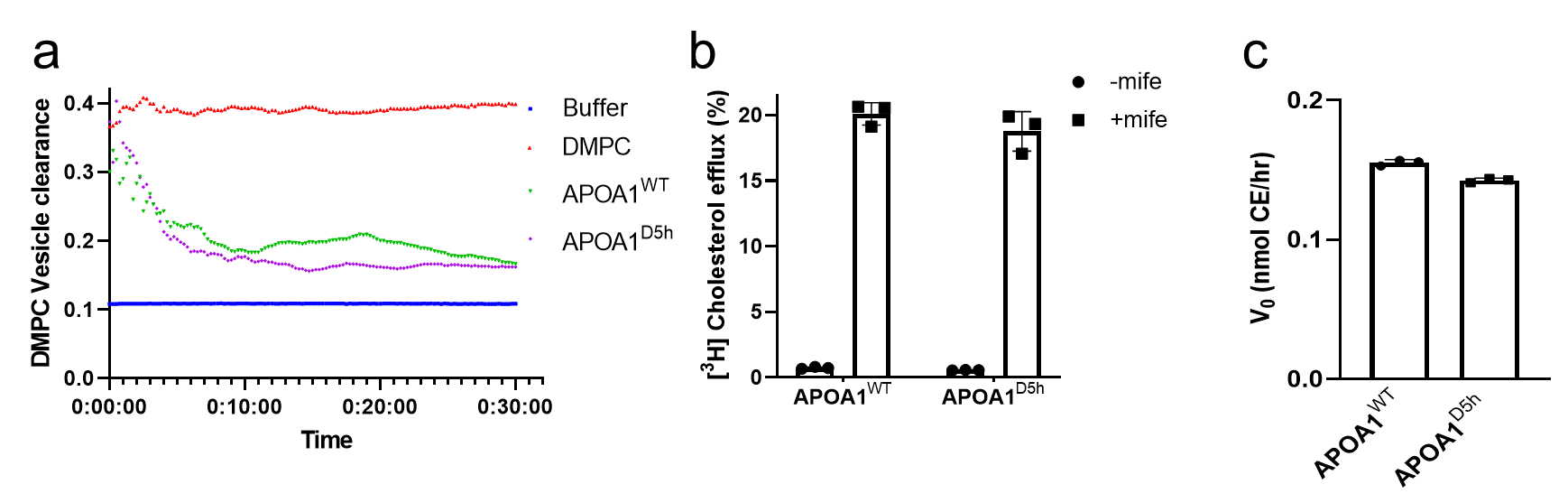


**Figure S3**: **Functional characterization of the purified APOA1^D5h^ mutant protein**. **a)** Ability of lipid-free APOA1 and mutant to clear multilamellar liposomes of dimyristoyl phosphatidylcholine (DMPC) at 24.5 °C. The liposomes remained suspended in buffer alone during the 30 min incubation (red trace) whereas WT APOA1 reorganized them to small discoidal particles that scatter less light at 325 nm. APOA1^D5h^ performed similarly to APOA1^WT^. Traces are representative of triplicate measurements. **b**) Ability of lipid free APOA1 and mutant to promote cholesterol efflux from baby hamster kidney cells that have been engineered to express human the ATP-binding cassette transporter, a critical cell surface protein that mediates physiological cholesterol efflux from cells upon the addition of mifepristone. In the absence of ABCA1 expression, little efflux of tritiated cholesterol was detected. However, upon ABCA1 induction, both forms of APOA1 were equally effective acceptors of cellular cholesterol. Data represents one experiment performed in triplicate. Error bars show 1 sample standard deviation. **c**) Ability of the mutants in rHDL particles to activate LCAT. rHDL particles generated with APOA1^WT^ and APOA1^D5h^ were of similar morphology, though rHDL^D5h^ exhibited a slight increase in particle diameter and cargo phospholipid (see manuscript **Fig. 4b,c**). Both particles were analyzed in our LCAT activity assay. There was a modest, although statistically significant due to the tightness of the error bars, reduction in activity with rHDL^D5h^. The experiment was performed in triplicate and is representative of 2 independent experiments with similar results. The error bars show 1 sample SD. Altogether; these data show that the addition of an extra copy of helix 5 had little impact on the functionality of APOA1 for the functions tested here.

**Supplement Figure S4**

**
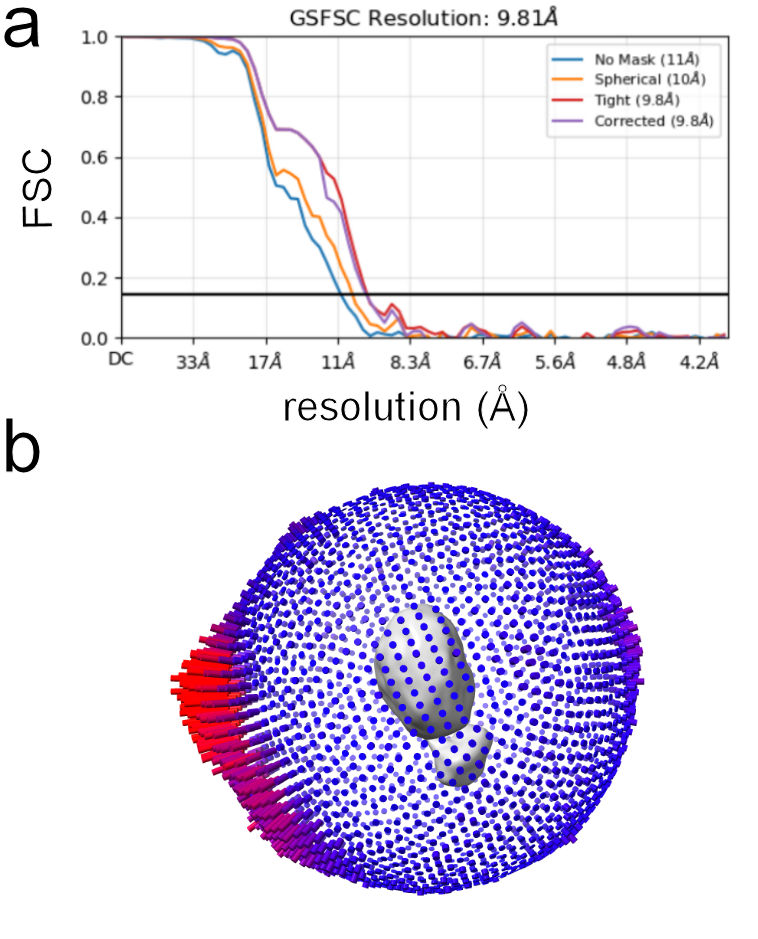
**

**Figure S4**: **CryoEM map parameters**. **a)** Fourier Shell Correlation (FSC) curves showing the correlation of two independent maps generated from two random halves of the data according to Chen et al. ^4^. The solid black line shows the threshold at which the estimated resolution was determined. **b**) 3-Dimensional representation of angular distribution viewed as a Euler plot.

**Supplement Figure S5**

**
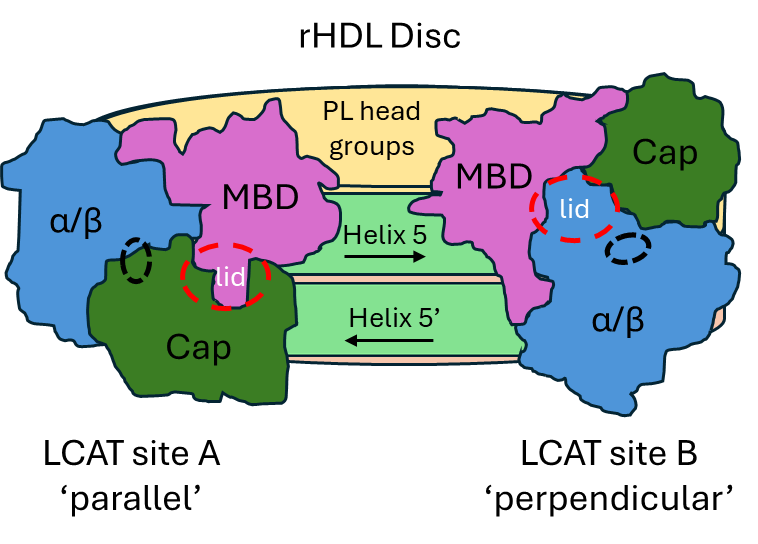
**

**Figure S5: Cartoon of LCAT positions with respect to the rHDL disc bilayer and speculations as to potential mechanistic implications.** A schematic of the docked structure shown in **Fig. 6** is shown approximately to scale. The top face of the rHDL phospholipid headgroup region is shown in yellow while the 5^th^ helices of the two resident APOA1 molecules are shown in lime green. The LCATs are colored with the α/β hydrolase domain in blue, the membrane binding domain in purple, and the cap domain in forest green. The approximate position of the lid regions for each LCAT are shown as dashed circles as they are behind the enzyme and interacting with the rHDL edge. The approximate location of the catalytic triad of each LCAT (Ser-181, Asp-345 and His-377) is shown by the black dotted circle (also on the back side of the enzyme and facing the rHDL particle). We can speculate on two possible explanations for the different orientations of LCAT observed. First, the two positions might represent different steps of the reaction cycle. For example, LCAT at site B is positioned so that its MBD interacts with the polar headgroups of the rHDL surface lipids potentially allowing for substrate PL and cholesterol to be pulled into the active site from the particle faces. The different orientation at site A may reflect a conformational change that facilitates a subsequent reaction step. A second possibility is that one of the orientations is an artifact of the experimental system. To reproducibly obtain enough LCAT-APOA1 complexes for structural study, we were forced to go above physiological ratios of LCAT to APOA1 in our EM and cross-linking studies. LCAT concentrations in normal human plasma range from 5-6 µg/ml whereas APOA1 is typically 1.5 mg/ml, a 500-fold molar excess vs. LCAT. Thus, our experimental conditions could have driven the formation of lower affinity and perhaps non-physiological complexes. Future studies will require specific mutagenesis of one APOA1 epitope vs the other and assessing the effect on the LCAT docking orientation as well as its activity.

**Supplement Figure S6**

**
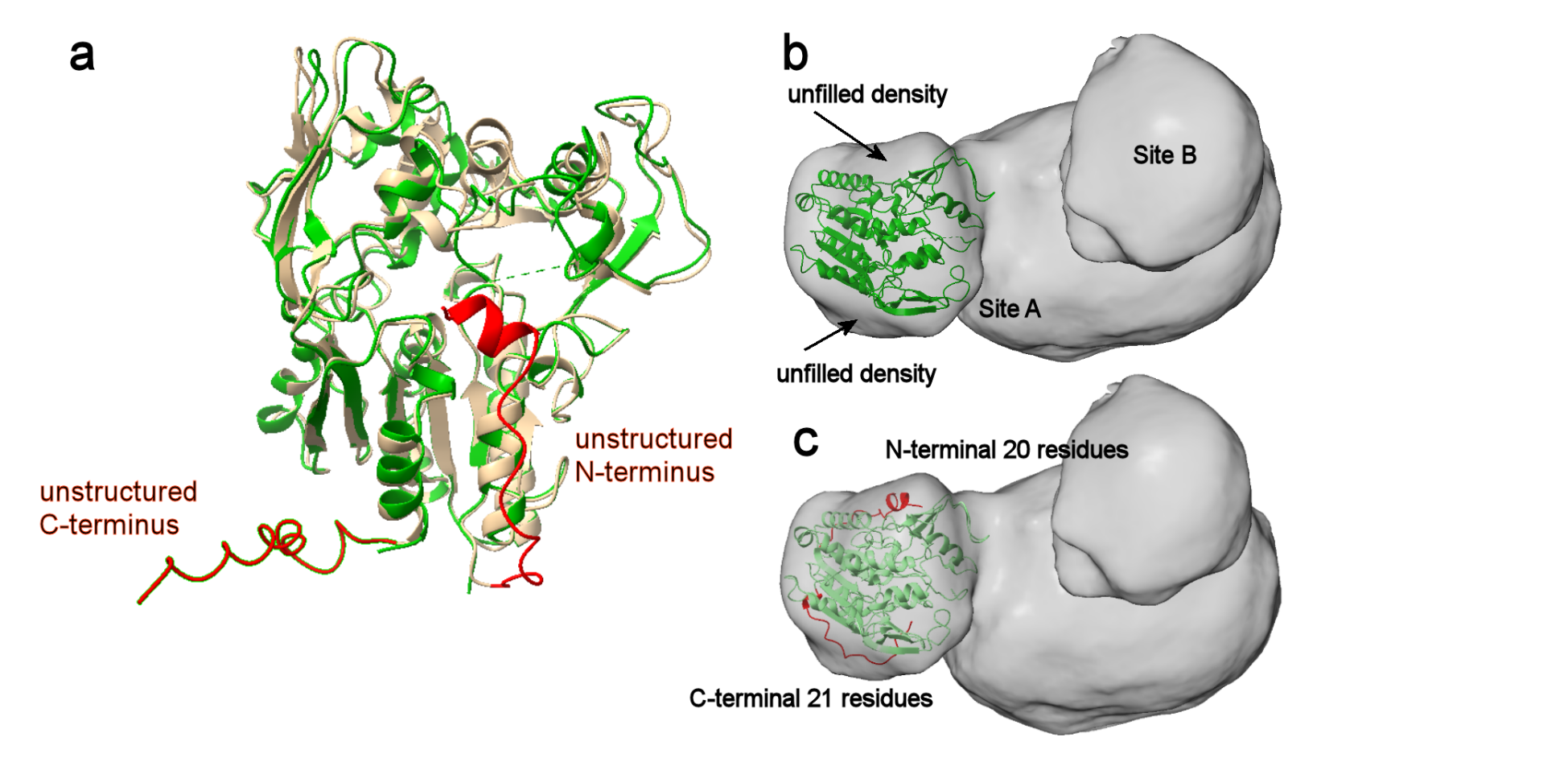
**

**Figure S6**: **Using AlphaFold 3 to model full-length LCAT using the cryo-EM envelope for binding site A**. Available crystal structures of LCAT have not been able to visualize the highly flexible N-terminus (20 residues) and C-terminus (21 residues) of human LCAT. After fitting LCAT to our cryo-EM envelope, we noticed two areas of unfilled density. This was most clear for the LCAT interacting with binding site A (parallel to the APOA1 belt) because this showed the highest consistency in the 2-D class averages. We used AlphaFold 3 to generate a putative structure of mature human LCAT (lacking its signal sequence) then we manually manipulated the model to fill the two density voids in the map. **a**) AlphaFold 3 ^5^ predicted structure (tan) overlaid on the crystal structure 4XWG (green). The N- and C-termini were modeled by AlphaFold as relatively unstructured strands will low confidence scores (red). **b**) Docked model of 4XWG LCAT structure on 100-2 rHDL particle with two prominent areas of unfilled density indicated. **c**) We performed a manual manipulation of the missing terminal domains guided by the cryo-EM density map and followed it with a simple energy minimization in Chimera 1.18. The N-terminal 20 residues loop back across the α/β hydrolase domain and contacts the LCAT cap domain. This puts the LCAT N-terminus in proximity to the rHDL particle, and it also suggests that the highly conserved N-terminus of mature LCAT (after signal peptide processing) might contribute to the active site pocket, which would explain its importance for activity. The C-terminus also crosses back across the α/β hydrolase domain on the opposite side, away from the HDL particle.

**References:**

1 Segrest, J. P., Jones, M. K., Shao, B. & Heinecke, J. W. An experimentally robust model of monomeric apolipoprotein A-I created from a chimera of two X-ray structures and molecular dynamics simulations. *Biochemistry* **53**, 7625-7640, doi:10.1021/bi501111j (2014).

2 Pourmousa, M. *et al.* Tertiary structure of apolipoprotein A-I in nascent high-density lipoproteins. *Proc Natl Acad Sci U S A* **115**, 5163-5168, doi:10.1073/pnas.1721181115 (2018).

3 Lima, D. B. *et al.* Characterization of homodimer interfaces with cross-linking mass spectrometry and isotopically labeled proteins. *Nat Protoc* **13**, 431-458, doi:10.1038/nprot.2017.113 (2018).

4 Chen, S. *et al.* High-resolution noise substitution to measure overfitting and validate resolution in 3D structure determination by single particle electron cryomicroscopy. *Ultramicroscopy* **135**, 24-35, doi:10.1016/j.ultramic.2013.06.004 (2013).

5 Abramson, J. *et al.* Accurate structure prediction of biomolecular interactions with AlphaFold 3. *Nature* **630**, 493-500, doi:10.1038/s41586-024-07487-w (2024).
